# Phenotypic Selection, Genetic Variation, and Genome-Wide SNP Fingerprinting of Typhoon-Surviving Candidate Individuals in *Casuarina equisetifolia*

**DOI:** 10.3390/plants15213320

**Published:** 2026-10-27

**Authors:** Huichang Xu, Yawen Tian, Jiayun Li, Huihua Zeng, Shuaitao Chen, Jiawei Li, Sen Nie, Gongfu Ye

**Affiliations:** 1 College of Forestry, Fujian Agriculture and Forestry University, Fuzhou 350002, China; xhc199809@163.com (H.X.);; 2 Fujian Academy of Forestry Sciences, Fuzhou 350012, China

**Keywords:** *Casuarina equisetifolia*, genetic variation analysis, whole-genome resequencing, SNP markers, genetic diversity analysis, DNA fingerprinting

## Abstract

*Casuarina equisetifolia* L. is a key pioneer tree species for coastal shelterbelt plantations in tropical and subtropical regions. However, long-term reliance on a limited number of superior clones has narrowed its genetic base and reduced stress resistance, which necessitates the selection of elite individuals and the construction of molecular identities to support precise germplasm identification. In this study, 108 ten-year-old half-sib families from 23 provenances were evaluated for four traits: tree height (H), diameter at breast height (DBH), volume (V), and wind-throw resistance grade (WTRG). Highly significant variation was detected among blocks, families, and block-by-family interactions for all traits (*p* < 0.001). Phenotypic and genotypic coefficients of variation ranged from 47.52–81.55% and 9.43–14.89%, respectively. Using combined selection, 29 elite individuals were identified, achieving genetic gains of 6.02%, 11.30%, 34.44%, and 3.84% for H, DBH, V, and WTRG, respectively. Whole-genome resequencing of these 29 individuals generated high-quality data, yielding 3,533,880 high-confidence SNP loci after initial filtering. After stringent quality control, 379 core SNP markers were retained, with a mean polymorphic information content (PIC) of 0.366 and a minor allele frequency (MAF) of 0.420, indicating their potential utility for individual discrimination. Based on these core SNPs, a DNA fingerprint database was constructed and integrated with QR code technology to create digital identity tags for each elite individual. Our results indicate that the tested *C. equisetifolia* population harbors considerable genetic variation and exhibits potential for genetic improvement. The established SNP-based fingerprinting system, together with the QR-code-enabled digital tagging platform, provides technical support for precise identification and germplasm management of elite genotypes.

## 1. Introduction

*Casuarina equisetifolia* L. is naturally distributed in tropical to subtropical coastal areas, including northern Queensland of Australia, southern Thailand, and Malaysia [1,2]. Owing to its rapid growth, well-developed root system, and strong ecological adaptability such as wind and sand resistance and salt tolerance, it has become one of the major pioneer tree species for the establishment of coastal shelterbelts and vegetation restoration on difficult coastal sites worldwide [3,4,5]. At present, the area of Casuarina plantations in southeastern coastal China and India has exceeded 300,000 hm^2^ and 500,000 hm^2^, respectively, playing a vital role in mitigating coastal natural disasters, improving coastal soils, and promoting ecological restoration [6,7]. However, long-term afforestation relying on a limited number of superior clones has led to a progressively narrowing genetic base and a marked decline in overall stand stress resistance, severely constraining the sustained provision of its ecological protection functions [8,9]. Meanwhile, the increasing frequency and intensity of storm surges and typhoons driven by global climate change have imposed greater environmental stress on existing germplasm resources [10,11]. Therefore, there is an urgent need to select breeding materials with enhanced stress resistance to improve the adaptability and stability of Casuarina plantations.

Sexual breeding and clonal utilization has long been the core strategy guiding genetic improvement of *C. equisetifolia* [12]. Since the 1980s, through international cooperation projects such as those between China and Australia and between China and France, China has successively introduced more than 70 provenances of *C. equisetifolia* from different countries and regions, and established systematic provenance trial forests in Fujian, Guangdong, and Hainan provinces [5,6]. Li [13] conducted early selection based on 42-month growth data from 109 families representing 23 provenances, and identified 28 fast-growing superior families as candidate materials for subsequent cross-breeding. Wei et al. [14] investigated growth traits of 4-year-old trial plantations involving 21 provenances at Xuwen, Guangdong, and confirmed significant differences among provenances in height, diameter at breast height, and volume, leading to the comprehensive selection of four superior provenances. However, despite considerable progress in germplasm evaluation and breeding population construction, systematic screening of elite individuals combined with their DNA fingerprints remains lacking for *C. equisetifolia*. There is currently no molecular identity system capable of rapidly and accurately identifying breeding materials, thereby constraining the intellectual property protection of elite genotypes and the promotion of improved varieties.

Single nucleotide polymorphism (SNP) markers have been widely applied in genetic diversity analysis and DNA fingerprinting of forest trees due to their advantages of high genetic stability, low detection cost, and high throughput [15,16]. Liu et al. [17] successfully constructed DNA fingerprints of 52 tea germplasm accessions using SNP markers, while Wan et al. [18] developed molecular identities for 187 macadamia germplasm accessions based on SNP markers. However, the application of this approach to elite individual identification and fingerprint construction in *C. equisetifolia* has not yet been reported. Therefore, this study examined 108 half-sib families from 23 provenances of *C. equisetifolia*, systematically measuring growth traits including tree height (H), diameter at breast height (DBH), and volume (V) in the 10-year-old trees. Taking advantage of the natural occurrence of super typhoon “Doksuri”, the wind-throw resistance grade (WTRG) of each individual tree was assessed to analyze the patterns of variation in growth traits and wind-throw resistance, and elite individuals were selected by combining both types of traits. Furthermore, whole-genome resequencing was performed on these elite individuals to assess their genetic diversity, screen core SNP loci, and construct DNA fingerprints. This study aims to provide technical support for the precise selection of elite *C. equisetifolia* individuals and the construction of their molecular identities, while also laying a germplasm foundation for broadening the genetic base and directionally breeding new wind-resistant germplasm.

yijk= μ+Bi+Fj+B×Fij+ℇijk∆Gg=S×h2x¯×100%φi

## 2. Results

### 2.1. Variation and Correlation Analysis of Growth Traits and Wind-Throw Resistance Grade

Four traits, H, DBH, V, and WTRG, were statistically analyzed and subjected to analysis of variance (ANOVA) for the tested *C. equisetifolia* population (Table 1). The results showed that the mean WTRG was 2.96, indicating that the tested population exhibited relatively strong wind resistance overall. The ANOVA results revealed that all four traits differed highly significantly among blocks, among families, and for the block-by-family interaction (*p* < 0.001). Among these sources of variation, the block effect exhibited the largest F-value for all traits, suggesting that environmental heterogeneity within the trial site was the primary factor driving phenotypic variation.

**Figure 1:**
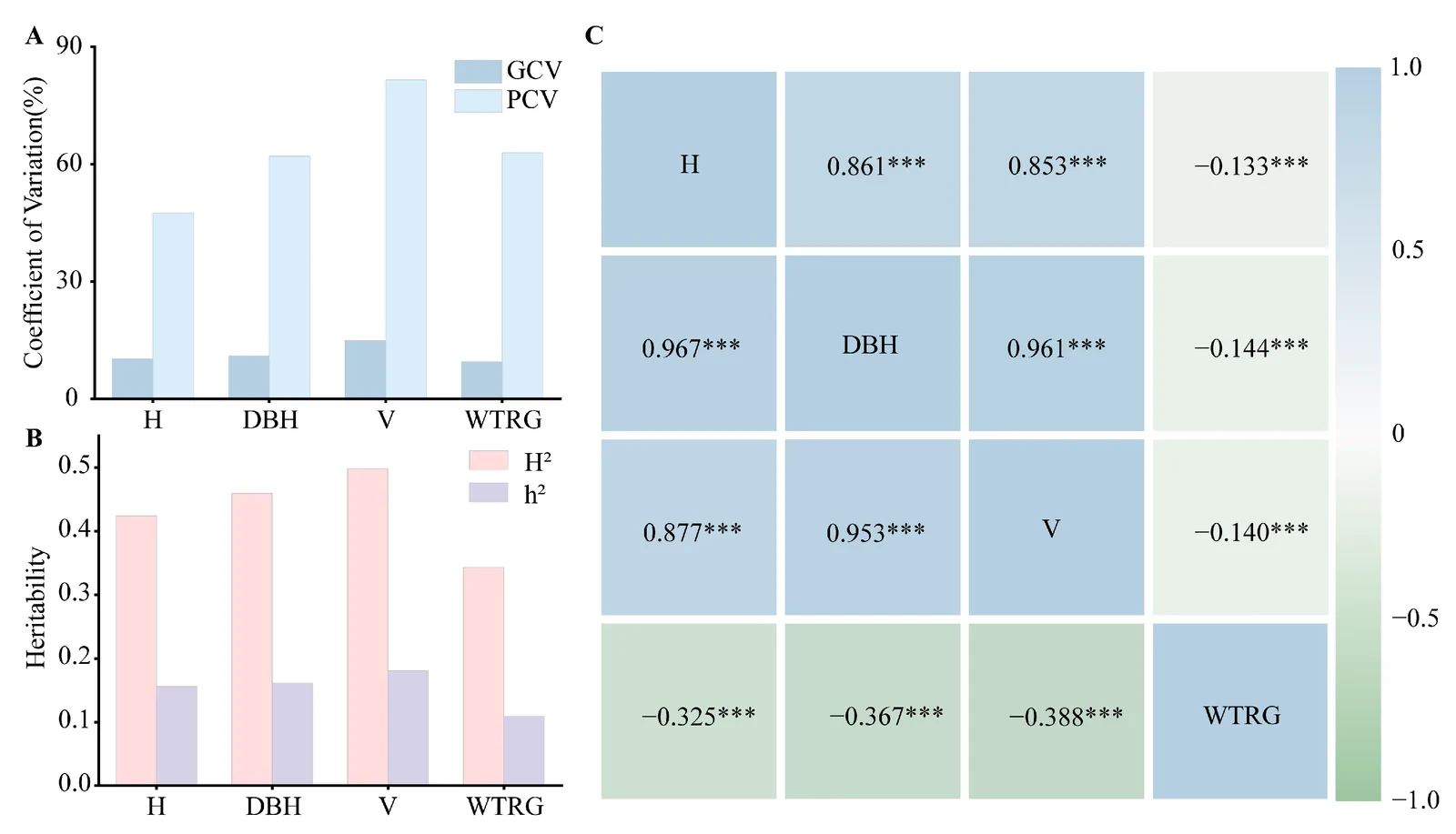
Genetic variation and correlation analysis of growth traits and wind-throw resistance grade. (**A**) Phenotypic coefficient of variation (PCV) and genotypic coefficient of variation (GCV) for growth traits. (**B**) Broad-sense heritability (H^2^) and narrow-sense heritability (h^2^) for growth traits and wind-throw resistance grade. (**C**) Phenotypic (upper triangle) and genetic (lower triangle) correlation coefficients among growth traits and wind-throw resistance grade. Notes: H: tree height; DBH: diameter at breast height; V: stem volume; WTRG: wind-throw resistance grade; “***” *p* < 0.001.

The PCV for the four traits of *C. equisetifolia* ranged from 47.52% to 81.55%, which were higher than the corresponding GCV (Figure 1A), indicating that phenotypic variation was predominantly driven by environmental effects, while the additive genetic component was relatively limited. Broad-sense heritability ranged from 0.3433 to 0.4989 (Figure 1B), representing a moderate level. Among the traits, V exhibited the highest value, 0.4989, whereas WTRG showed the lowest, 0.3433, reflecting differences in the degree of genetic control across traits. Narrow-sense heritability was generally low, ranging from 0.1093 to 0.1809, further confirming that the additive genetic variance accounted for a relatively small proportion of the total phenotypic variance. Both phenotypic and genetic correlations among growth traits (H, DBH, and V) were highly significantly and positive (*p* < 0.001) (Figure 1C), with the highest correlation observed between DBH and stem volume (phenotypic r = 0.961, genetic r = 0.953). In contrast, wind-throw resistance grade showed significant negative phenotypic and genetic correlations with H, DBH, and V.

### 2.2. Selection of Elite Individuals

**Figure 2:**
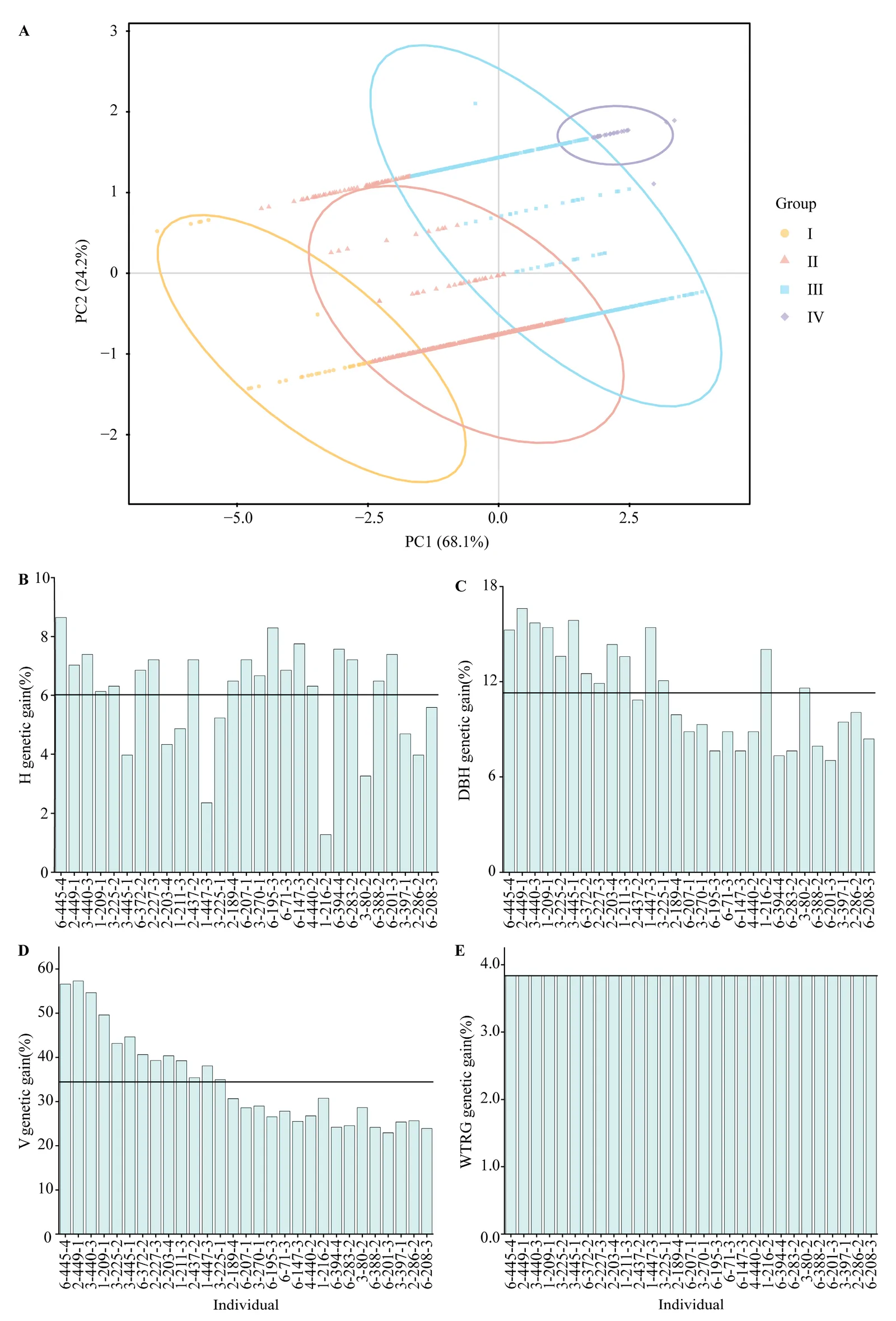
Cluster analysis of the tested population and evaluation of genetic gains in elite individuals. (**A**) Principal component analysis (PCA) score plot of the tested population based on growth traits. Polygons in different colors represent the four clusters (Group I–IV) identified by hierarchical clustering. (**B**–**E**) Genetic gain of elite individuals selected for H (**B**), DBH (**C**), V (**D**), and WTRG (**E**), respectively. Horizontal lines indicate the mean genetic gain of the population. H: tree height; DBH: diameter at breast height; V: stem volume; WTRG: wind-throw resistance grade.

Comprehensive evaluation of 1050 individual trees was conducted using the Breeding Value Multi-trait Evaluation Method and cluster analysis, with tree height, DBH, stem volume, and wind-throw resistance grade as assessment indicators. When the Euclidean distance was set to 5, the tested population could be divided into four clusters (Figure 2A). Cluster I contained 36 individuals, and exhibited positive genetic gains for all four traits: 6.47%, 12.46%, 39.92%, and 2.15% for H, DBH, V, and WTRG, respectively; accordingly, this cluster was classified as fast-growing individuals. Cluster II comprised 536 individuals, with genetic gains of 1.94%, 2.49%, 4.87%, and 2.09%, respectively. Cluster III contained 454 individuals, all of which showed negative genetic gains (−2.38%, −3.40%, −7.85%, and −2.37%). Cluster IV consisted of 24 individuals, also with negative genetic gains (−6.81%, −9.08%, −16.00%, and −7.10%). Clusters III and IV were classified as slow-growing individuals (Table S1).

From the best-performing Cluster I, 29 individuals with a wind-throw resistance grade of 4 were further selected as elite individuals. Their mean tree height, DBH, stem volume, and wind-throw resistance grade were 12.04 ± 0.99 m, 18.00 ± 2.04 cm, 0.1368 ± 0.03 m^3^, and 4, respectively, exceeding the overall population means by 3.37 m, 7.41 cm, 0.0626 m^3^, and 1.04, respectively. The mean genetic gains for these traits were 6.02%, 11.30%, 34.44%, and 3.84%, respectively (Figure 2B–E). The selected elite individuals showed notable improvements in volume growth while retaining a wind-throw resistance grade of 4, thereby providing candidate germplasm materials for subsequent breeding and genetic improvement of fast-growing and wind-resistant *C. equisetifolia* varieties.

### 2.3. Genome Resequencing Quality and SNP Variation Detection

Raw sequencing data from the 29 elite individuals were subjected to rigorous filtering and quality control, and the resulting high-quality clean reads were aligned to the *C. equisetifolia* reference genome. Data statistics (Table S2) showed that the average clean bases per sample was 4.17 Gb. Q20 and Q30 values ranged from 97.30–98.21% and 93.08–95.21%, with mean values of 97.86% and 94.42%, respectively, indicating high base-calling quality across all samples. The GC content ranged from 36.63% to 40.26% (mean 37.58%), reflecting normal base composition of the sequenced genomes. Regarding alignment performance, the average mapping rate of the 29 samples to the reference genome was 93.64%, with a mean sequencing depth of 13.69×. The genome coverage rates at 1×, 5×, and 10× depth averaged 95.19%, 86.60%, and 62.33%, respectively. These metrics indicate that the resequencing data had satisfactory coverage and alignment quality, providing a basis for subsequent genome-wide detection of variants (SNPs and InDels).

**Figure 3:**
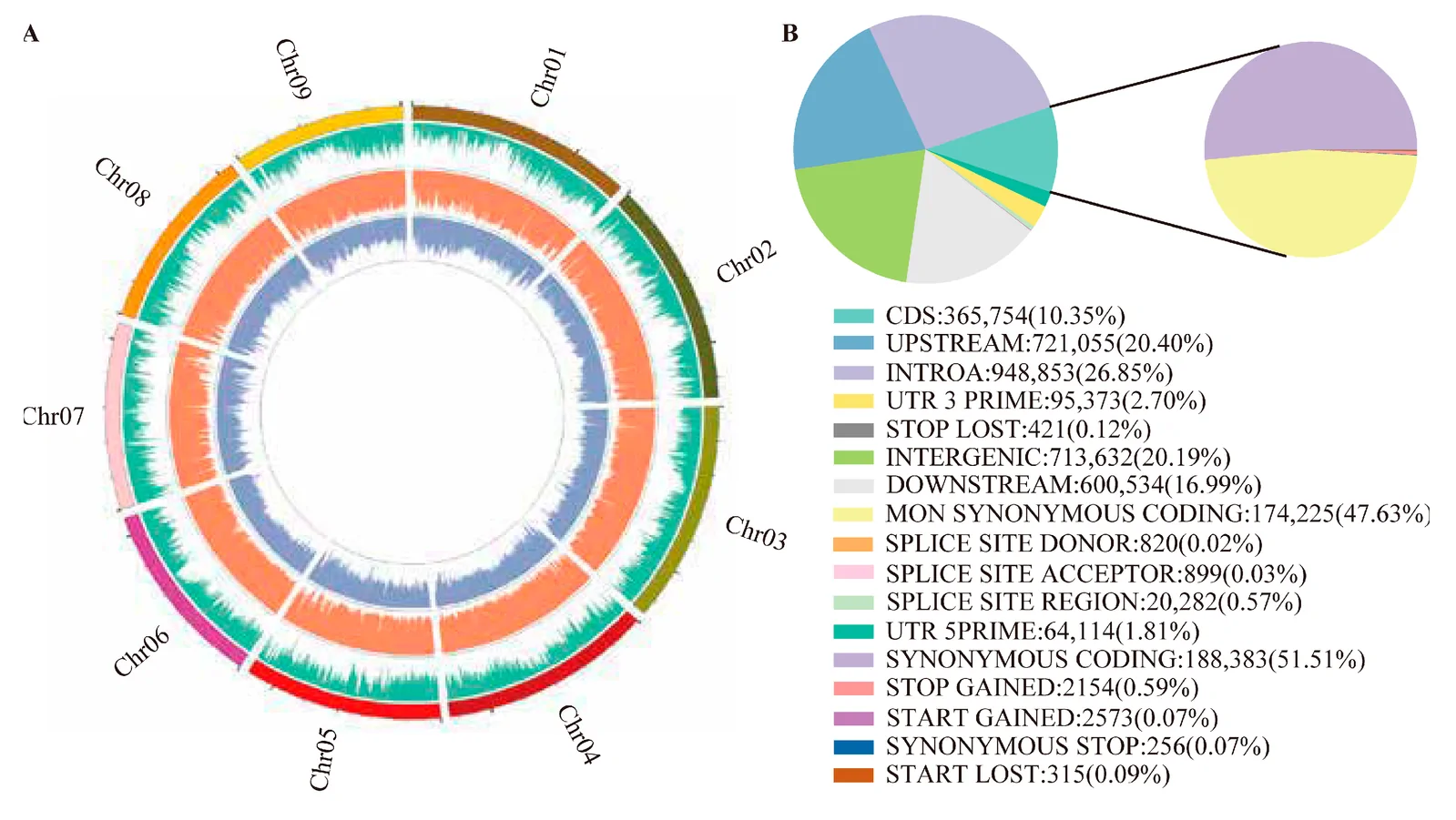
Genome-wide distribution of SNPs and InDels and functional annotation of SNPs in *C. equisetifolia*. (**A**) Chromosomal distribution of gene, SNP and InDel densities (from the outer to inner rings are chromosome number and physical scale, gene density, SNP density and InDel density, respectively). (**B**) Functional annotation distribution of genome-wide SNPs across different genomic regions (the left panel shows the proportions across the whole genome, and the right panel shows the proportions of mutation types within core coding regions).

Based on the high-quality alignment results, variant detection and hard filtering were performed using GATK, yielding a total of 3,533,880 high-quality SNPs and 618,489 InDels across the genome, indicating abundant genomic variation among the 29 elite individuals. Statistical analysis of nucleotide substitution types for the high-quality SNPs revealed an average transition/transversion ratio (Ti/Tv) of 2.05, which is within the range commonly observed in plant genomes and supports the reliability of the identified SNP loci. As shown in the chromosome-level density distributions of genes, SNPs, and InDels (Figure 3A), these three features exhibited non-uniform distribution across the genome, with certain chromosomal regions displaying differential variation densities. Functional annotation of SNPs (Figure 3B) indicated that variant loci were predominantly enriched in non-coding regions, with variations in intronic, intergenic, and upstream/downstream regions collectively accounting for 84.43% of all SNPs. Only 10.35% of SNPs were located in coding sequences (CDS). Within the CDS region, synonymous mutations (51.51%) and non-synonymous mutations (47.63%) occurred at a nearly 1:1 ratio. The genomic distribution pattern of InDels was generally consistent with that of SNPs. The variant dataset described above provides a substantial resource for subsequent screening of core SNP markers and construction of DNA fingerprints.

### 2.4. Population Genetic Structure Analysis

**Figure 4:**
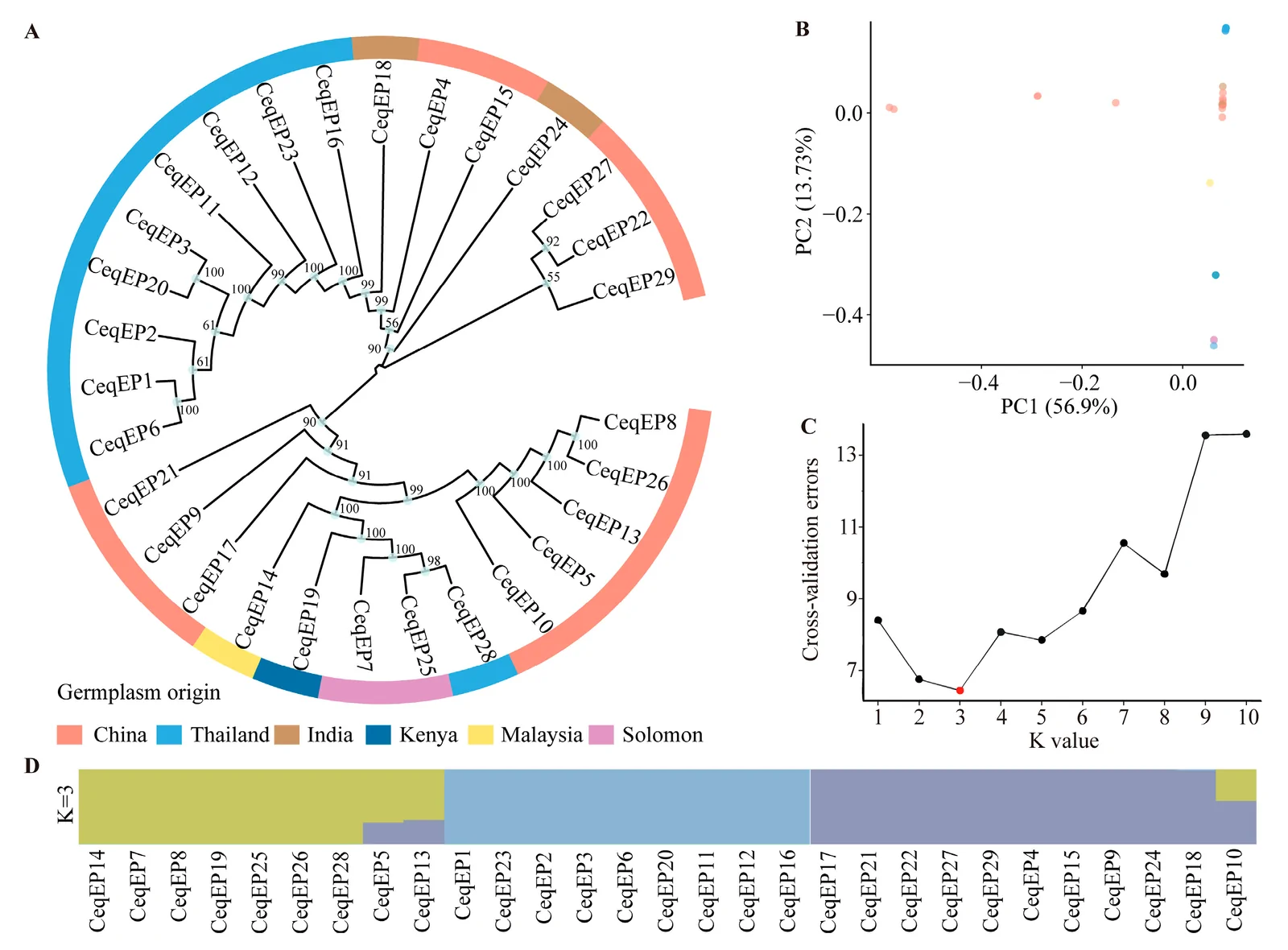
Genetic structure analysis of *C. equisetifolia*. (**A**) Phylogenetic tree constructed based on genome-wide SNPs. Numbers at nodes represent bootstrap support values (based on 1000 replicates), displayed as percentages. (**B**) Principal component analysis (PCA) plot. Dots are colored by geographical origin, as in (**A**). (**C**) Cross-validation errors for different K values. (**D**) Population structure at K = 3. Each vertical bar represents an individual, and colors indicate the inferred ancestry proportions.

Based on 3,533,880 high-quality SNP markers, the population genetic background of the 29 elite individuals was analyzed. The phylogenetic tree (Figure 4A) showed that the tested individuals were distributed across multiple branches, with some clustering patterns that appeared to be associated with their geographical origins. Principal component analysis (Figure 4B) was consistent with this differentiation trend: PC1 and PC2 explained 56.9% and 13.73% of the total variation, respectively, with a cumulative explanatory rate of 70.63%. While the samples were generally concentrated in the two-dimensional scatter plot, certain individuals displayed a dispersed distribution, suggesting genetic heterogeneity within the population. Population structure analysis indicated that the lowest cross-validation error was achieved when K = 3 (Figure 4C,D). Accordingly, the 29 individuals were assigned to three subpopulations. The first subpopulation comprised nine samples with diverse geographical origins, including Kenya, Solomon Islands, China, Thailand, and Malaysia. The second subpopulation also contained nine samples, all originating from Thailand, showing a notable geographical aggregation. The third subpopulation consisted of eleven samples, of which two were from India and nine from China.

Collectively, the results of the phylogenetic tree, principal component analysis, and population structure analysis suggested that the 29 elite individuals had diverse genetic backgrounds and that genetic structure was associated with geographical origin to some extent. These results provide a basis for subsequent screening of core SNP markers.

### 2.5. Core SNP Screening and Genetic Diversity Analysis

To construct DNA fingerprints suitable for elite individual identification in *C. equisetifolia*, a stringent screening strategy was adopted that considered data completeness, uniformity of genomic distribution, minor allele frequency (MAF) and polymorphic information content (PIC), as well as stability of flanking sequences. Through this process, 379 core SNP loci were ultimately selected from the high-quality SNP dataset. Starting from the high-quality SNP dataset, the stepwise filtering procedure (see Section 4.4) retained 173,194 SNPs after basic quality control (100% call rate, MAF ≥ 0.20, HWE *p* > 0.01), 65,214 SNPs after applying the PIC ≥ 0.35 threshold, and 54,926 SNPs after removing markers with neighboring variants within 100 bp. Ultimately, 379 core SNP loci were selected following linkage disequilibrium thinning of the remaining SNP set.

Genetic diversity evaluation (Table 2) showed that the observed number of alleles for all 379 loci was 2.00, confirming that they were all bi-allelic variants. The mean effective number of alleles was 1.937, indicating a relatively balanced distribution of allele frequencies within the population. The mean observed heterozygosity and expected heterozygosity were 0.377 and 0.483, respectively. The mean values of Nei’s diversity index, Shannon’s diversity index, and PIC reached 0.492, 0.676, and 0.366, respectively, with an overall mean MAF of 0.420. Collectively, these parameters indicate that the strictly screened core SNP set was highly polymorphic and genetically diverse within the tested population, which supports its use for individual identification in this study. These characteristics provide a basis for DNA fingerprint construction and germplasm identification in *C. equisetifolia.*

### 2.6. DNA Fingerprint Construction and QR Code Generation for Elite Individuals

**Figure 5:**
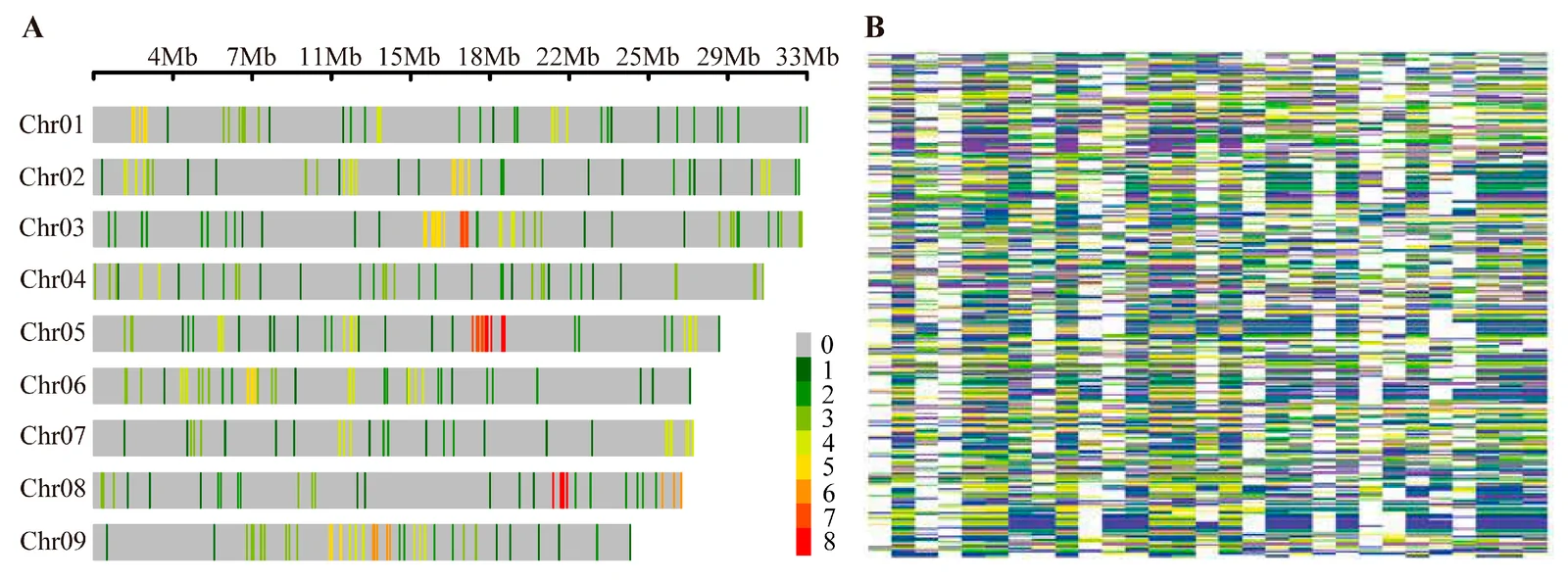
Distribution of core SNPs on chromosomes and DNA fingerprints of elite individuals in *C. equisetifolia*. (**A**) Distribution of 379 core SNPs on the nine chromosomes (the blue short lines indicate the physical positions of core SNPs on each chromosome, and the vertical axis represents the physical distance). (**B**) DNA fingerprint heatmap of 29 elite individuals based on 379 core SNPs (each row represents a candidate SNP site, and each column represents one sample information; yellow, green, blue, and purple represent C/C, A/A, T/T, and G/G, respectively, gray represents missing data, and white represents heterozygous sites).

The physical distribution of the 379 core SNP loci across the nine chromosomes of the *C. equisetifolia* genome was analyzed. The results showed that these loci were distributed on all nine chromosomes, with a minimum physical distance of 552 bp between adjacent loci and no obvious local clustering, suggesting that the selected markers exhibit a sparse and genome-wide coverage pattern (Figure 5A and Table S3). Based on these core loci, DNA fingerprints were constructed for the 29 elite individuals (Figure 5B). The fingerprint profiles revealed that each individual exhibited a highly specific genotypic combination, with clear differentiation among individuals. This indicates that the selected core SNP set possesses strong discriminatory power, enabling precise distinction among the 29 tested individuals.

To facilitate the storage, retrieval, and practical application of fingerprint information, the individual-specific genotype data were further encoded and converted into corresponding identity QR codes (Table S4), thereby enabling rapid reading and digital management of genetic information for *C. equisetifolia* germplasm. Each QR code encoded a plain-text string containing the individual identity (name/ID) and the complete genotype profile across all 379 core SNP loci (see Section 4.5). The order of loci in the fingerprint code was consistent with the physical arrangement of the 379 core SNPs on the chromosomes, which serves as the decoding basis for the QR codes.

## 3. Discussion

Variation and heredity constitute the prerequisite and foundation for genetic improvement [19,20]. In this study, the phenotypic coefficient of variation of the *C. equisetifolia* population ranged from 47.52% to 81.55%, which was higher than that reported by Hu et al. [21] for seedling-stage phenotypic variation in *C. equisetifolia*. This relatively high level of phenotypic variation may be closely associated with the complex site conditions of the experimental site. The trees were continuously subjected to multiple stresses such as wind damage and salt spray during growth, which may have amplified their phenotypic plasticity. It should be noted that ANOVA showed that the block effect had the largest F-value for all traits, indicating that environmental heterogeneity or microenvironment within the trial site was the primary source of phenotypic variation. Because wind damage was assessed only after a single typhoon, it remains unclear whether wind exposure was uniform among plots. If blocks differed in wind shielding, topography, edge effects, or soil moisture, WTRG results could be biased by non-genetic factors. Moreover, extreme events such as typhoons acted as strong selective pressures, further intensifying phenotypic differentiation among surviving individuals, collectively contributing to the elevated phenotypic coefficient of variation in the tested population [22,23]. In contrast, the genotypic coefficient of variation ranged from 9.43% to 14.89%, indicating a low-to-moderate level, suggesting that a substantial portion of the phenotypic variation originated from genotype-by-environment interaction and non-genetic noise, while the additive genetic component remained relatively constrained. This finding was consistent with the trend of heritability estimates for this population.

In this study, the heritability of growth traits at age 10 ranged from 0.1093 to 0.4989, which was generally low to moderate, and was lower than the heritability estimates for 4-year-old *C. equisetifolia* reported by Zeng et al. [24] This indicates that the heritability of growth traits decreased with increasing stand age. The possible explanation is that competition for nutrients and growing space among individual trees was relatively weak at age 4, whereas with increasing age, neighbor interference gradually intensified, deepening the mutual influence among individuals. Consequently, tree growth became more constrained by environmental factors, leading to an increase in genotype-by-environment interaction variance and thus a decline in heritability [25]. In addition, the pronounced and largest block effect in this study may also have reduced heritability estimates by inflating environmental variance. Therefore, the heritability values here should be interpreted as estimates under the specific microenvironmental conditions of this trial, rather than as fixed parameters. Overall, the low-to-moderate genotypic coefficient of variation and heritability may be consistent with the hypothesis that the tested *C. equisetifolia* population has experienced directional selection under long-term environmental stress, with the additive genetic component potentially tending to converge. However, no direct evidence of directional selection was obtained in the present study, and this interpretation therefore remains speculative and requires further verification. If this hypothesis is supported by future selection-signature analyses and long-term multi-site trials, future breeding efforts should not only continue selecting candidate individuals from existing families but also broaden the genetic base through the introduction of new provenances or hybridization, so as to enhance the genetic diversity and long-term improvement potential of the population.

The correlation coefficient quantitatively characterizes the covariation among different traits and serves as an important indicator for revealing the intrinsic associations and degree of interaction among target traits [26]. In this study, both the phenotypic and genetic correlation coefficients between V and DBH were the highest among all trait pairs, which is consistent with the results reported by Zeng et al. [24] for growth traits in 4-year-old *C. equisetifolia*. This suggests that DBH can serve as an effective indirect selection criterion for volume improvement, whereby selecting individuals with larger DBH can simultaneously achieve volume gain. The high genetic correlation also implies that the genes controlling these two traits may be tightly linked or pleiotropic, facilitating joint selection in breeding practice and thereby improving selection efficiency. Genetic gain is an important indicator for evaluating the effectiveness of artificial selection [27]. In this study, using a 3% selection intensity and a WTRG = 4 as the threshold, a total of 29 elite individuals were selected, achieving genetic gains of 6.02%, 11.30%, 34.44%, and 3.84% for H, DBH, V, and WTRG, respectively. However, it should be noted that the present study was based on only a single typhoon event; therefore, the inferred selective effects and the associated phenotypic differentiation may not fully reflect the long-term consequences of recurrent typhoon disturbances. In future studies, we will conduct observations across multiple sites and multiple typhoon events, record the spatial distribution of wind exposure and wind damage, and use mixed or spatial models to separate genetic from non-genetic effects.

In this study, 29 elite individuals were selected from 1050 candidates, corresponding to a selection proportion of approximately 2.76%. Although such intensive selection can yield substantial short-term genetic gains, the potential cost to population genetic diversity should not be overlooked. If the next breeding generation were derived solely from these 29 individuals through random intercrossing, the effective population size would decrease markedly, increasing the risks of inbreeding accumulation, genetic drift, and loss of rare alleles, which could in turn weaken the population’s long-term adaptive potential to pests, diseases, climate change, and future typhoon stress. Of particular concern is that WTRG selection in this study was based on wind-damage performance after a single typhoon, and that the block effect was the largest source of variation across all traits. Under such conditions, selected individuals may partly reflect plot-level microenvironmental or wind-exposure differences rather than stable genetic resistance, further amplifying selection bias. Therefore, future breeding should not rely solely on random intercrossing among a small number of elite individuals. A more prudent strategy would be to combine within-family and among-family selection, retain a sufficient number of candidate families and individuals, control relatedness during mating, conduct multi-trait and multi-site selection, and use molecular markers to continuously monitor genetic diversity and inbreeding levels. In addition, new provenances should be introduced, or hybridization should be conducted, in a planned manner to broaden the genetic base, thereby balancing short-term genetic gain with long-term breeding potential.

These elite individuals were mainly derived from China and Thailand, accounting for 44.83% and 34.48% of the total (Table S5), respectively. The highest proportion of Chinese-origin individuals may be attributed to the fact that the parental materials of the tested germplasm have developed strong ecological adaptability to China’s coastal site conditions through long-term introduction and local domestication. The Thai-origin materials also exhibited outstanding performance, likely because both the trial site and the Thai source regions are influenced by monsoon circulation, characterized by high temperature and humidity. Such similar climatic conditions provided a favorable growing environment for the introduced materials [28]. Therefore, in future collection and introduction of *C. equisetifolia* germplasm, greater attention could be given to materials from Thailand and climatically similar regions, although their adaptability and promotion potential under different ecological conditions still need to be evaluated through regional trials.

SNP markers have become an ideal tool for genetic research of germplasm resources due to their abundance, wide genomic distribution, and high stability [15,16]. In this study, whole-genome resequencing was performed on 29 elite individuals for SNP identification, and a total of 379 core SNP loci were screened. These core loci exhibited an observed number of alleles of 2.000, an effective number of alleles of 1.937, and a Shannon’s diversity index of 0.676, all of which were higher than those reported by Huang et al. [29] and Xu et al. [30], who employed AFLP and ISSR markers in 18 *C. equisetifolia* provenances and 51 clones, respectively. This difference may be attributed to the higher detection efficiency and broader genomic coverage of SNP markers compared with AFLP and ISSR markers, enabling them to more sensitively capture genetic variation information across different loci and thus yield higher diversity parameter estimates [31]. Nevertheless, these estimates were derived from a discovery panel of only 29 individuals, and the core loci were selected for polymorphism within this panel. They may therefore not represent genome-wide or species-level genetic diversity and should not be extrapolated to the broader breeding population.

The 379 core SNPs are primarily intended for individual identification and are sufficient to distinguish the 29 selected individuals. However, their discriminatory power for a much larger future breeding population is likely to be limited. Because the panel was developed from a small number of individuals, it may suffer from ascertainment bias, favoring loci that are polymorphic in the discovery set while underrepresenting rare alleles, low-frequency variants, and variation in genomic regions not captured by the sampled individuals. When applied to hundreds or thousands of individuals, including close relatives, full-sibs, or clones, the panel may not provide adequate resolution for individual identification or kinship assignment, nor is it suitable for population genetic structure analysis, genome-wide association studies, or genomic selection. In addition, these 379 core SNPs have not been validated in an independent sample set, so their discriminatory power and reliability at the population level remain to be confirmed. Future work should therefore expand germplasm sampling, validate this panel in independent populations, and evaluate its probability of identity, probability of sibship exclusion, minor allele frequency, and linkage disequilibrium. If necessary, higher-density SNP arrays or larger marker sets based on whole-genome resequencing should be developed. These efforts should be integrated with long-term multi-site phenotypic evaluation to identify core clones with desirable phenotypes and stable genetic performance.

## 4. Materials and Methods

### 4.1. Plant Materials and Study Site

In March 2013, the Fujian Academy of Forestry and the Research Institute of Tropical Forestry, Chinese Academy of Forestry, collaboratively sowed seeds of 108 half-sib families from 23 provenances of *C. equisetifolia* by family in the nursery of Huian Chihu state-owned protection forest farm. These materials comprised previously selected Chinese elite germplasm and introduced germplasm from Australia, India, Vietnam, Kenya, Malaysia, Thailand, Vanuatu, Benin, and Solomon Islands (Table S5). In April 2014, a provenance/family trial plantation was established using a randomized complete block design with six blocks. Each plot consisted of a single row of four trees, spaced at 2 m × 2 m.

The study site is located at Huian Chihu state-owned protection forest farm, Fujian province (118°55′ E, 24°35′ N), approximately 1 km from the coastline, at an elevation of 9 m above sea level. The area has a mean annual temperature of 20–22 °C, mean annual precipitation of 1200–1600 mm, and mean annual evaporation of approximately 2000 mm, with abundant sunlight throughout the year. The soil is coastal sandy loam with moderate fertility.

### 4.2. Phenotypic Trait Investigation and Data Processing

In May 2023, growth traits including tree height (H), diameter at breast height (DBH), and stem volume (V) were measured for each individual tree in the 10-year-old half-sib family trial plantation of *C. equisetifolia*. The V was estimated according to the volumetric equation by Li [32].

Super typhoon “Doksuri”, the fifth typhoon of 2023, made landfall along the coast of Jinjiang city, Fujian province, on 28 July 2023. Upon landfall, it weakened from a super typhoon to a severe typhoon, with maximum sustained winds of 50 m/s near its center. The trial site was approximately 50 km in straight-line distance from the landfall point and about 40 km from the nearest point of the post-landfall track. Affected by typhoon “Doksuri”, the trial plantation suffered varying degrees of wind-throw. Accordingly, a supplementary survey was conducted in September 2023 to record the wind-throw status of every individual tree in the trial plantation. Wind-throw resistance grade (WTRG) was classified into four levels according to the following criteria [33]: Grade 1, trunk broken or leaning angle > 45°; Grade 2, leaning angle between 30° and 45°; Grade 3, leaning angle between 15° and 30°; Grade 4, leaning angle < 15° or no leaning.

Linear mixed models were fitted using the lme4 package [34] in R 4.4.2 (https://www.r-project.org). The full statistical model for each trait was

*y_ijk_* = *μ* + *B_i_* + *F_j_* + (*B* × *F*)*_ij_* + *ℇ_ijk_*

where *y_ijk_* is the observation of the *k*-th tree in the *i*-th block and *j*-th family; *μ* is the overall mean; *B_i_* is the random effect of the *i*-th block; *F_j_* is the random effect of the *j*-th family; (*B* × *F*)*_ij_* is the random interaction effect between block and family; and *ℇ_ijk_* is the residual error.

Phenotypic and genotypic coefficients of variation, broad-sense heritability, and narrow-sense heritability were calculated from the estimated variance components. The genetic gain from selection (∆*G_g_*, %) was computed as

∆Gg=S×h2x¯×100%

where *S* is the selection differential (the difference between the mean of the selected individuals and the population mean), *h*^2^ is the single-plant heritability, and $\bar{x}$ is the overall phenotypic mean of the trait.

The breeding value multi-trait evaluation method was implemented by calculating a composite score for each individual according to the following model:

$$\varphi_{i}\;=\;\sqrt{\sum_{j=1}^{n}\frac{{Χ}_{\mathrm{ij}}}{{Χ}_{\mathrm{jmax}}}}$$

where φ*_i_* is the composite score of the *i*-th individual; *Χ_ij_* is the mean value of the *j*-th trait for the *i*-th individual; and *Χ_jmax_* is the optimal value (maximum) of the *j*-th trait across all tested individuals.

The optimal value for each trait was defined as the maximum value across all tested individuals.

### 4.3. DNA Extraction, Resequencing, and SNP Detection

The 29 elite individuals used for whole-genome resequencing were selected from the 1050 trees evaluated in the trial plantation. The selection was based on the breeding value multi-trait evaluation method combined with cluster analysis, with tree height (H), diameter at breast height (DBH), stem volume (V), and wind-throw resistance grade (WTRG) as assessment indicators (see Section 4.2). At a Euclidean distance of 5, the tested population was divided into four clusters; Cluster I, comprising 36 individuals with positive genetic gains for all four traits, was identified as the best-performing group. From Cluster I, 29 individuals with a WTRG of 4 (i.e., leaning angle < 15° or no leaning after typhoon “Doksuri”) were selected as elite individuals. Detailed selection results are presented in Section 2.2.

Genomic DNA was extracted from 29 elite individuals using the modified CTAB method. DNA concentration and purity were assessed with a NanoDrop 2000 (Thermo Fisher Scientific, Waltham, MA, USA). Qualified DNA samples were subjected to library construction and whole-genome resequencing. Sequencing libraries with an insert size of approximately 350 bp were constructed using the NEBNext^®^ Ultra^TM^ DNA Library Prep Kit for Illumina (New England Biolabs, Ipswich, MA, USA) following the manufacturer’s protocol. The libraries were sequenced on an Illumina NovaSeq 6000 platform using the SBS (sequencing by synthesis) chemistry with 150-bp paired-end reads.

Raw sequencing data were processed using fastp (https://github.com/OpenGene/fastp, accessed on 1 April 2026) to remove adapters, reads with >10% unknown bases (N), and low-quality reads, yielding clean data. Clean reads were aligned to the chromosome-level reference genome of *Casuarina equisetifolia* published by Zhang et al. [35] (GenBank accession: GCA_028551475.1) using bwa-mem2 (https://github.com/bwa-mem2/bwa-mem2, accessed on 1 April 2026), sorted with samtools v1.9 (http://www.htslib.org/), and SNP calling was performed using GATK v3.8 (https://gatk.broadinstitute.org/, accessed on 1 April 2026). Initial filtering employed GATK’s hard-filtering strategy with thresholds: QD < 2.0, FS > 60.0, MQ < 40.0, MQRankSum < −12.5, QUAL < 30.0, and ReadPosRankSum < −8.0.

### 4.4. Population Genetic Structure Analysis and Core SNP Selection

Using the high-quality SNPs, a neighbor-joining phylogenetic tree was constructed with MEGA X (https://www.megasoftware.net/, accessed on 1 April 2026) under the p-distance model and 1000 bootstrap replicates. Principal component analysis was performed using smartpca in EIGENSOFT v6.0 (https://github.com/DReichLab/EIG, accessed on 1 April 2026), and population genetic structure was analyzed with Admixture v1.22 (https://dalexander.github.io/admixture/, accessed on 1 April 2026).

Subsequently, a stepwise filtering procedure was applied using VCFtools v0.1.15 (https://vcftools.github.io/man_latest.html, accessed on 1 April 2026) to develop a core SNP panel for individual identification. (1) Basic filtering was performed with VCFtools under stringent completeness criteria, retaining only biallelic SNPs with a 100% call rate (no missing data), a minor allele frequency (MAF) of at least 0.20, and a Hardy–Weinberg equilibrium (HWE) *p*-value > 0.01, which yielded 173,194 SNPs. The MAF threshold of ≥0.20 aligns with the stringent filtering criteria employed in individual-identification SNP panel development, where markers with low MAF are excluded to ensure discriminative power [36]. (2) SNPs with a polymorphic information content (PIC) < 0.35 were discarded, and 65,214 SNPs were retained. The PIC threshold of ≥0.35 was chosen to ensure moderate-to-high marker informativeness, which is more stringent than the conventional criterion that a PIC > 0.25 indicates an informative marker. (3) To prevent tight physical clustering of markers, only SNPs with no other variants located within 100 bp upstream or downstream were retained (i.e., the distance to the nearest neighboring retained SNP exceeded 100 bp), leaving 54,926 SNPs. (4) Finally, to obtain a compact panel with independent markers, the remaining SNPs were thinned using PLINK (https://www.cog-genomics.org/plink/, accessed on 1 April 2026) with a fixed random seed to break linkage disequilibrium, and 379 core SNPs were ultimately retained. These 379 core SNPs were located on all nine chromosomes, with a minimum physical distance of 552 bp between adjacent loci, suggesting a sparse and genome-wide coverage generally compatible with DNA fingerprinting construction.

### 4.5. DNA Fingerprint Construction and QR Code Generation

Genotype data of the 29 elite individuals at the 379 core SNP loci were extracted and encoded to generate individual-specific DNA fingerprints. Each fingerprint code consisted of the genotypes at the core SNP loci arranged in a fixed order, with the two alleles at each locus separated by a slash (e.g., A/A, G/T, A/G). The encoded fingerprint information was then converted into QR codes using qrencode software (https://fukuchi.org/works/qrencode/, accessed on 1 April 2026). Each QR code embedded two fields: the individual name (e.g., “Name: Ceq1”) and the corresponding fingerprint code (e.g., “fingerprint code: A/A G/T A/G …”). This design allowed the individual identity and its SNP genotype profile to be retrieved directly by scanning. It was adopted to enable rapid, digitized, and visual identification of germplasm individuals, reduce manual recording errors, and support precise germplasm management.

## 5. Conclusions

This study evaluated the phenotypic and genetic variation characteristics of 108 half-sib families from 23 provenances of *C. equisetifolia*. The results showed that the phenotypic coefficient of variation ranged from 47.52% to 81.55%, and the genotypic coefficient of variation ranged from 9.43% to 14.89%. Using a 3% selection intensity and a WTRG = 4 as the criteria, 29 elite individuals were selected, with genetic gains of 6.02%, 11.30%, 34.44%, and 3.84% for H, DBH, V, and WTRG, respectively. Furthermore, based on whole-genome resequencing, 379 core SNP loci were identified from these 29 elite individuals, and a DNA fingerprint database was constructed. In combination with QR code technology, a digital identity tag was assigned to each elite individual. This study provides an integrated approach for elite individual selection that combines phenotypic evaluation, genetic assessment, and molecular identification, and offers a reference for variety breeding and germplasm identification of *C. equisetifolia*.

## Figures and Tables

**Table 1:** Mean values and analysis of variance for phenotypic traits of the tested population.

Traits	Min	Max	Mean ± SD	Source of Variation	df	MS	*F-*Value	*p-*Value
Tree height/H	1.800	13.900	8.64 ± 2.32	Block	5	124.580	45.401	<0.001
				Family	107	10.052	3.663	<0.001
				Block × Family	535	5.607	2.043	<0.001
Diameter at breast height/DBH	1.500	24.000	10.59 ± 3.92	Block	5	133.182	14.305	<0.001
				Family	107	34.813	3.739	<0.001
				Block × Family	535	16.400	1.761	<0.001
Stem volume/V	0.0003	0.256	0.0467 ± 0.0373	Block	5	0.018	23.214	<0.001
				Family	107	0.003	3.936	<0.001
				Block × Family	535	0.002	2.116	<0.001
Wind-throw resistance grade/WTRG	1.000	4.000	2.96 ± 1.38	Block	5	18.753	13.882	<0.001
				Family	107	3.153	2.334	<0.001
				Block × Family	535	1.942	1.438	<0.001

**Table 2:** Statistics of genetic diversity parameters for candidate core SNP loci.

Index	Total	Min–Max (Ave)
Number of alleles (*Na*)	758.000	2.000–2.000 (2.000)
Effective number of alleles (*Ne*)	734.141	1.859–2.000 (1.937)
Observed heterozygosity (*Ho*)	127.793	0.241–0.759 (0.377)
Expected heterozygosity (*He*)	183.202	0.462–0.500 (0.483)
Nei’s diversity index (I)	186.416	0.470–0.509 (0.492)
Shannon’s diversity index (I′)	256.344	0.655–0.693 (0.676)
Polymorphic information content (PIC)	138.887	0.355–0.375 (0.366)
Minor allele frequency (MAF)	0.420	-
Number of markers (*N*)	379.000	-

## Data Availability

The raw whole-genome resequencing data of the 29 individuals of *C. equisetifolia* generated in this study have been deposited in the Genome Sequence Archive (GSA) under accession number CRA051007.

## Supplementary Materials

The following supporting information can be downloaded at https://www.mdpi.com/article/10.3390/plants15213320/s1, Table S1: Genetic gains of H, DBH, V, and WTRG for *Casuarina equisetifolia* individuals in different clusters. Table S2: Quality control of whole-genome sequencing data for 29 elite individuals of *Casuarina equisetifolia*. Table S3: Genotype matrix of the 379 core SNP loci across the 29 elite *Casuarina equisetifolia* individuals. Table S4: DNA fingerprint QR codes for 29 elite individuals of *Casuarina equisetifolia*. Table S5: Provenance information of *Casuarina equisetifolia*.
