# Supplementary material for: Phenotypic Selection, Genetic Variation, and Genome-Wide SNP Fingerprinting of Typhoon-Surviving Candidate Individuals in Casuarina equisetifolia
Source: Plants (Basel). 2026 Oct 27;15(21):3320. doi: 10.3390/plants15213320 (PMC13766072; doi:10.3390/plants15213320)
Supplement: Supplementary file 1 [file plants-15-03320-s001.zip › Table S4 DNA fingerprint QR codes for 29 elite individuals of Casuarina equisetifolia.pdf]

Table S4 DNA fingerprint QR codes for 29 elite individuals of *Casuarina equisetifolia*

Elite individual ID	DNA fingerprints ID	QR	Raw data encoded in the QR code
6-445-4	Ceq1(CeqEP1)		Name: Ceq1; fingerprint code: A/A G/T A/G A/G A/G A/G C/T G/G A/A G/G C/C T/T C/C C/C T/T G/G C/T A/A G/G A/A A/A A/G C/C C/T G/G C/C A/A A/A C/C C/T G/G A/C C/T G/G G/G C/C T/T G/G A/A A/G A/C A/A A/A C/C C/C T/T C/C C/G C/C C/C T/T A/A A/A C/C T/T C/C G/G C/C A/A G/T C/T A/A G/G A/A A/A A/A A/G A/G T/T G/G G/G C/C G/G C/C T/T G/G G/G C/T T/T C/C T/T C/C A/A A/C T/T T/T T/T C/C C/C A/A T/T G/G A/A T/T T/T A/A A/A G/G T/T A/A A/A T/T A/A A/A G/G T/T T/T G/G G/G G/G C/C T/T T/T A/A C/C A/A C/C G/G C/C A/T C/C G/G T/T A/A C/C C/C C/C G/G A/G A/T C/T G/G A/A A/A G/G A/A G/G A/G A/C G/G G/G T/T T/T C/T A/A T/T C/T G/G T/T A/G A/G A/G G/G A/T A/C A/A T/T C/C C/T A/A G/G A/A G/T C/T C/C C/T T/T G/G A/G T/T A/A C/C G/G A/A G/G C/G A/A A/G C/C G/G A/T G/G C/C G/T C/C T/T G/G C/G A/G A/A T/T G/T T/T G/G C/C C/C A/A G/G A/G C/T A/A T/T T/T G/G A/A T/T C/C T/T T/T C/C T/T T/T A/A A/A C/C C/C A/T A/A G/G G/G T/T A/A C/C A/G T/T A/G A/A G/G G/G T/T C/C T/T A/A C/C A/A A/A A/A A/A G/G G/G G/G A/A A/A C/C T/T G/G T/T C/C G/G G/G A/T T/T C/C T/T T/T A/G G/G G/G T/T A/A T/T A/A C/T G/G C/C G/G C/C A/A G/G C/C C/C G/G A/A C/T A/A A/A A/A T/T T/T C/C C/T A/A C/C A/A C/C G/G A/G C/T A/C A/A A/A T/T A/A C/C T/T C/C C/T C/C A/A T/T T/T A/A A/G A/A C/C A/G A/A A/A G/G A/A G/G A/A C/T T/T A/A G/G G/G G/G C/T A/A A/T A/A G/G C/C C/T C/C T/T G/G C/T T/T C/T A/A T/T G/G G/G A/T A/A G/G C/C T/T A/A C/T T/T A/A T/T T/T A/A T/T T/T T/T G/G G/G T/T G/G T/T G/G T/T G/G T/T A/A T/T A/A C/T T/T C/T C/T C/T T/T C/C G/G G/G C/G A/A T/T A/G T/T C/T C/T A/T
2-449-1	Ceq2(CeqEP2)		Name: Ceq2; fingerprint code: A/A G/G A/G G/G A/G A/G C/T C/G A/G G/G C/T C/C C/C C/C T/T G/G C/T A/A G/G A/A A/A A/G C/C C/T G/G C/C A/A A/A C/C C/T G/G A/C C/T G/G G/G C/C T/T A/A A/A A/A A/A A/A A/G C/C C/T C/T C/C C/C C/C C/C T/T A/A A/A C/C T/T C/C G/G C/C A/A G/T C/C A/A G/G A/A A/A A/A A/G A/G T/T A/G G/G C/G G/G C/C T/T G/G G/G C/C T/T C/C T/T C/C A/A A/A T/T T/T T/T C/C C/C A/A T/T G/G A/A T/T T/T A/A A/A G/G T/T A/A A/A T/T A/A A/A G/G T/T T/T G/G G/G G/G C/C T/T T/T A/A C/C A/A C/C G/G C/C A/T C/C G/G A/T A/A C/T C/C C/C G/T A/G A/T T/T G/G A/A A/A A/G A/A G/G A/G A/C A/G G/G T/T T/T C/T A/A C/T C/C A/G T/T G/G G/G A/G G/G A/A C/C A/G C/T C/C C/T A/A A/A A/A G/T C/C T/T C/T C/T A/G A/G C/T A/A C/T A/G A/A G/G G/G A/A G/G C/C G/G A/A A/G C/C G/G T/T C/T G/G C/C A/G A/A T/T G/T T/T G/G C/C C/C A/A G/G G/G C/T A/A T/T T/T G/G A/A T/T C/C T/T T/T C/C T/T T/T A/A A/A C/C C/C T/T G/G G/G C/G C/T A/T T/T A/G A/T G/G A/A G/G G/G T/T C/C T/T A/A C/C A/A A/A A/G A/A G/G G/G G/G A/A A/A C/T T/T G/G T/T C/C G/G A/G A/A T/T C/C T/T T/T A/A G/G G/G T/T A/A T/T A/A T/T G/G C/C G/G C/T A/A G/G C/C C/C G/G A/A T/T G/G A/A A/A T/T T/T C/C C/C A/G A/A A/A T/T A/A A/G C/T A/C A/A A/A T/T A/A C/C T/T C/C C/T C/C A/A T/T T/T A/G A/A A/A C/T A/G A/A A/A G/G A/A G/G A/A C/T T/T A/A G/G G/G G/G C/T A/A T/T A/A G/G C/C C/C C/C T/T G/G T/T T/T C/T A/A T/T G/G G/G A/T A/A G/G C/C T/T A/A C/T T/T A/A T/T T/T A/A T/T T/T T/T G/G G/G T/T G/G T/T G/G T/T G/G T/T A/A T/T A/A C/T T/T C/C T/T T/T A/T C/C G/G G/G C/C A/T C/C A/G T/T C/T C/T T/T
3-440-3	Ceq3(CeqEP3)		Name: Ceq3; fingerprint code: A/A G/T A/G A/G A/G A/A C/T G/G A/G A/G C/C T/T C/T C/C T/T G/G C/T A/A G/G A/A A/A A/G C/C T/T G/G C/C A/A A/A C/C C/T G/G A/C C/C G/G G/G C/C T/T A/G G/G A/A C/C A/A A/G C/C C/C T/T C/T C/C C/C C/T T/T G/G G/G C/C T/T C/C G/G C/C A/A G/G C/C A/A G/G A/A A/A A/A A/G A/G T/T G/G G/G C/G A/G C/C T/T A/G A/G C/C T/T C/C C/T C/G A/G A/A T/T T/T T/T C/C C/T A/A T/T G/G A/A T/T T/T A/A A/A G/G T/T A/A A/A T/T A/A A/A G/G C/T T/T G/G G/G G/G C/C T/T T/T A/A C/C A/A C/C G/G C/C A/T C/C G/G T/T A/A C/T C/C C/C G/T A/G A/T C/C G/G A/A A/C A/G A/A G/G A/G A/C G/G G/G T/T T/T C/C A/A C/T T/T G/G T/T A/G A/G A/G G/G A/T A/C A/G T/T T/T T/T A/A A/G A/A T/T C/C C/T C/C C/C A/A G/G C/T A/G C/T A/G A/A C/C C/G A/A G/G C/C G/G T/T A/G C/C G/G C/T C/T G/G C/C A/G A/T T/T G/T T/T G/G C/C C/C A/A G/G G/G T/T A/A T/T T/T G/G A/A T/T C/C T/T T/T C/C T/T T/T A/A A/A C/C C/C T/T A/G C/C C/G T/T A/A C/C A/G A/T A/A A/G G/G G/G T/T C/C T/T A/A C/C A/A A/A A/G A/A G/G G/G G/G A/A A/A C/T T/T G/G T/T C/C G/G A/A T/T T/T C/C T/T T/T A/G G/G G/G T/T A/A T/T A/C C/T G/G C/C A/A C/C A/A G/G C/G C/C C/G A/C C/T A/A A/A A/A C/T T/T C/C C/T A/G A/C A/A T/T A/G A/G T/T A/C A/A A/A C/T A/G C/C T/T C/C T/T C/C A/A T/T T/T A/G A/A A/T C/C G/G A/A A/A G/G A/A G/G A/G C/T T/T A/C C/G G/G G/G T/T A/A A/A A/A G/G C/C C/T A/C T/T G/G C/T T/T C/T A/A T/T G/G G/G A/A A/G G/G C/C T/T A/A C/T T/T A/A T/T C/T A/A T/T T/T T/T G/G G/G T/T G/G T/T G/G T/T G/G T/T A/A T/T A/A C/T T/T T/T C/C C/C A/A C/C G/G G/G C/G A/A T/T G/G T/T C/C C/C A/T

1-209-1	Ceq4(CeqEP4)	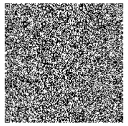	Name: Ceq4; fingerprint code: A/T G/G G/G G/G A/A A/A T/T C/G A/G G/G T/T T/T C/C C/C A/T G/T C/T A/C G/G A/A A/G A/G C/T T/T G/G C/C A/G A/G T/T T/T A/G A/A T/T G/G G/G C/C C/T G/G G/G G/G C/C A/G A/G C/T C/C T/T C/C C/C C/C C/T T/T A/G G/G T/T C/C C/G A/G C/T A/G G/G C/C A/A C/G A/A A/A A/A A/A A/A T/T A/A A/A C/G A/G G/G G/T G/G A/G C/C A/A A/C T/T C/C A/G A/C C/T C/T A/T C/T C/T A/A C/T A/G A/G C/T C/T A/G A/C A/G C/T A/G A/T C/T A/G A/G A/A C/C C/T G/G A/G A/G C/T C/T C/T A/G A/C A/T A/C A/G C/T A/T C/T G/T A/A A/G C/T C/T A/A G/T A/G T/T C/T A/G A/G A/A G/G A/G A/G A/G A/C A/G G/G T/T T/T C/C C/C C/T C/T A/G T/T A/A G/G A/G G/G A/A C/C A/G C/T C/T C/T A/A A/G A/G G/G C/T C/T T/T C/C A/A G/G T/T A/A C/C G/G G/G C/G C/C A/A G/G T/T G/G T/T G/G C/T T/T T/T C/C G/G C/C A/G A/T C/T G/T C/T G/G C/C C/T C/C G/G A/G C/T A/G A/T G/T A/G A/T C/T C/G C/T T/T C/G C/T C/T A/T A/G C/T C/T T/T A/G C/G C/G C/T A/A C/C A/A A/A G/G A/G C/G G/G T/T C/C T/T C/C A/A G/G A/G A/G A/A G/G G/G A/G A/G A/G C/T A/T A/G T/T C/C G/G G/G A/T T/T C/T C/T T/T A/G A/G C/G T/T A/C G/T A/A C/C G/G C/C A/G C/T A/G A/G C/G C/T C/G A/C T/T A/A G/G G/G C/C C/C T/T T/T G/G C/C A/C C/T A/G A/G C/C A/C A/T A/G C/T G/G C/T C/T C/T C/T C/T A/G A/T T/T G/G A/G A/T C/T A/A G/G A/A A/G A/C A/G G/G T/T G/T A/C G/G G/G A/G C/T A/G A/T A/C A/G C/T T/T A/C T/T A/G C/T C/T C/C A/G A/T A/G C/G T/T A/A G/T C/C T/T A/G C/T T/T A/A T/T C/T A/T C/T C/T C/C A/G G/T C/T A/G C/T C/G A/T G/T G/T A/G C/T A/A C/T T/T C/C C/T C/T A/A A/C C/G A/G C/C A/T C/T G/G A/T T/T T/T A/T
3-225-2	CeqEP5	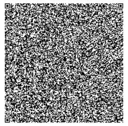	Name: CeqEP5; fingerprint code: A/T G/G A/G A/G G/G A/A C/T C/C G/G A/G T/T C/T C/T C/G A/T G/T C/T A/C A/G A/C A/G A/G T/T C/C A/G C/T G/G G/G C/T C/T A/G A/C C/T C/C A/G C/G C/T A/A A/G G/G A/A A/G G/G C/T C/T C/T C/C C/G C/G C/C G/T A/G A/G C/T C/T C/G A/G C/T A/G T/T C/T A/G C/G A/G A/G A/T A/G G/G G/T A/A A/G C/G A/G C/G G/T G/G A/A C/T A/T C/C C/C C/G A/G A/C C/T T/T A/T C/T T/T A/G C/T G/G A/G C/T C/T G/G A/C A/G C/C G/G A/T C/T A/G A/G A/A C/T C/C A/A A/A G/G C/T T/T C/T A/A A/A A/A A/C G/G C/T A/T C/T G/T A/T A/G T/T C/T C/C G/T A/A A/T C/C A/A G/G A/C A/G A/A A/G A/A A/C A/A A/G C/T G/T C/T A/C C/T C/T G/G C/T A/G A/A G/G A/G A/T A/C A/G C/T T/T C/T A/G G/G A/G G/T C/T C/C C/T C/T A/G A/A C/C A/G T/T A/A G/G G/G G/G G/G A/G C/T A/G A/T A/A C/T G/T C/T C/T A/G C/G A/G A/T C/C G/G C/C A/G C/T C/T A/C G/T A/G C/T A/A A/T G/G A/G A/T C/T C/G C/T T/T C/C C/T C/T A/T A/G C/T T/T A/T A/G C/G C/C C/C A/T T/T G/G A/T A/A G/G C/C C/G C/T C/T C/T A/C A/C A/G A/G G/G A/C C/G G/T G/G A/G A/G C/T A/T A/G A/T C/T G/T A/A A/T C/T C/T C/T C/T G/G A/G C/G C/T A/C G/G A/C C/T G/T C/G A/A C/T G/G A/G C/G C/T C/G A/C C/T A/A A/G A/G T/T C/T C/T T/T A/G A/C A/C C/T A/G A/A C/T C/C T/T G/G T/T A/G C/T C/T C/T C/T C/T A/G A/T C/T A/A A/A T/T C/T A/G A/G A/C A/G A/C A/G A/A C/C G/T A/C C/G T/T A/A C/T G/G T/T A/A A/A T/T C/C A/C C/T A/G T/T C/T C/C A/G A/T A/G C/G A/T A/G G/T C/T G/T A/A C/T C/C A/C C/T C/T T/T C/T C/C C/T A/A G/T C/T A/G C/T C/G A/T T/T G/T A/G C/T A/G C/T A/T T/T C/T C/T A/T A/A C/G A/G C/G T/T C/C A/A A/T C/T C/T A/T
3-445-1	Ceq5(CeqEP6)	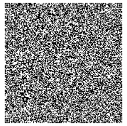	Name: Ceq5; fingerprint code: A/A G/T A/G A/G A/G A/A C/T G/G A/G A/G C/C C/T T/T C/C T/T G/T T/T A/A G/G A/A A/A A/G C/T C/T G/G C/C A/A A/A C/C C/C G/G A/C T/T G/G G/G C/C T/T G/G A/G A/A A/C A/A A/A C/C C/C T/T C/C C/G C/C C/T T/T A/A A/A C/C C/T C/C G/G C/C A/A G/G C/T A/G G/G A/A A/G A/A A/A A/A T/T G/G A/G C/G A/A C/C T/T G/G G/G C/T T/T C/C T/T C/C A/A A/A T/T T/T T/T C/C C/C A/A T/T G/G A/A T/T T/T A/A A/A G/G T/T A/A A/A T/T A/A A/A G/G T/T T/T G/G G/G G/G C/C T/T T/T A/A C/C A/A C/C G/G C/C A/T C/C G/G A/T A/A C/T C/C C/C G/T A/G A/T C/T G/G A/A A/A G/G A/A G/G A/G A/A A/G G/G T/T T/T C/T A/C C/T C/C A/G T/T A/G G/G A/G G/G A/T C/C A/G T/T T/T T/T A/A G/G A/A T/T C/T C/T C/T T/T A/A A/G T/T A/A C/T A/G A/A C/G G/G A/A A/G C/C G/G A/T A/G C/T G/G C/T T/T G/G C/G A/G A/A T/T G/T T/T G/G C/C C/C A/A G/G G/G C/T A/A T/T T/T G/G A/A T/T C/C T/T T/T C/C T/T T/T A/A A/A C/C C/C T/T A/A C/G C/G C/T T/T C/T A/G A/T A/A A/G G/G G/G T/T C/C T/T A/A C/C A/A A/A A/A A/A G/G G/G G/G A/A A/A C/T T/T G/G T/T C/C G/G G/G A/A T/T C/C T/T T/T G/G G/G G/G T/T A/A T/T A/A C/T G/G C/C A/A C/T A/A G/G C/C C/C G/G A/A C/C A/G A/A A/A T/T C/T C/T C/C G/G A/A A/A T/T A/G A/G T/T C/C A/A A/A C/T A/G C/C T/T C/C C/T C/C A/A T/T T/T G/G A/G A/A C/C A/G G/G A/A G/G A/A G/G G/G C/T T/T A/C G/G G/G A/G C/T A/A A/T A/A G/G C/C C/T C/C T/T G/G C/T T/T C/T A/A T/T G/G G/G A/T A/A G/G C/C T/T A/A C/T T/T A/A T/T C/T A/A T/T T/T T/T G/G G/G T/T G/G T/T G/G T/T G/G T/T A/A T/T A/A C/T T/T C/T T/T C/T A/A C/C G/G G/G C/C A/A C/T A/G T/T C/T C/T A/T

6-372-2	CeqEP7	Name: CeqEP7; fingerprint code: A/T T/T G/G A/A G/G G/G C/C G/G A/G A/G T/T C/T T/T G/G A/A G/G T/T C/C A/A C/C G/G G/G T/T C/C A/A T/T G/G G/G T/T C/C A/A A/A C/C C/C A/A G/G C/C A/A A/A A/G A/C G/G A/A T/T T/T C/C C/C G/G G/G C/C G/G A/A G/G T/T C/C G/G A/A T/T G/G T/T T/T G/G C/C G/G G/G T/T G/G G/G G/G G/G G/G G/G G/G G/G G/G A/G A/A T/T A/A A/A C/T G/G G/G C/C C/C C/C A/A T/T C/C G/G C/C A/A A/G C/C C/C A/G C/C A/A C/C G/G T/T C/C G/G G/G A/A C/C C/C A/G A/A A/A T/T C/C C/C G/G A/A T/T A/A A/A T/T A/T T/T T/T A/T G/G T/T C/T A/A T/T G/G T/T T/T A/A G/G C/C A/A A/A A/G A/G A/C A/A A/A C/C G/G T/T C/C C/T T/T A/G C/C G/G A/A A/G A/A T/T A/A G/G C/C C/C C/C G/G G/G G/G G/G T/T C/C T/T C/C A/G G/G C/C G/G T/T A/A A/G G/G G/G G/G A/G C/T A/A A/A A/A T/T T/T T/T C/C A/A G/G A/A T/T T/T G/G C/C A/A C/T T/T C/C T/T G/G T/T G/G A/A G/G A/A T/T C/C G/G C/C C/C G/G C/C C/C T/T G/G T/T T/T T/T A/A G/G C/C C/C T/T T/T G/G T/T A/A G/G C/G C/C C/C T/T C/C C/C A/A G/G G/G A/A C/C C/C T/T A/A G/G G/G C/T A/A A/A A/A T/T T/T A/A A/A C/T T/T C/C C/C A/A A/A C/C C/C C/C G/G C/C T/T T/T G/G A/A C/C G/G G/G G/G T/T C/C C/C C/C G/G G/G G/G C/C C/C T/T T/T G/G C/C C/C C/C G/G G/G T/T A/A T/T A/A C/T A/A T/T C/C T/T T/T T/T G/G A/A C/C A/A A/A T/T C/C G/G A/G C/C A/A C/C A/A G/G T/T G/G C/C C/C T/T G/G C/C A/A A/A C/C A/G T/T C/C C/C C/C A/A C/T C/C C/C G/G A/A A/A C/C T/T A/G T/T T/T T/T G/G C/C C/T C/C C/C C/T T/T C/C C/C C/C A/A T/T C/C A/A C/C C/C A/A T/T G/G G/G C/C G/G C/C A/A T/T T/T T/T T/T A/C C/C A/A G/G A/A C/C A/A A/A T/T T/T T/T
2-227-3	CeqEP8	Name: CeqEP8; fingerprint code: A/A G/G A/A A/A G/G A/A C/C C/C G/G A/A T/T C/T T/T G/G A/A G/T C/C C/C A/A C/C A/A G/G T/T C/C A/A T/T G/G G/G C/C C/C A/A C/C C/C C/C A/A G/G C/C A/A G/G G/G A/A G/G G/G T/T T/T C/C C/C G/G G/G C/C G/G G/G G/G T/T C/C C/C A/A T/T G/G T/T T/T G/G C/C G/G G/G T/T G/G G/G G/G A/A G/G G/G G/G C/C G/G G/G A/A T/T T/T C/C C/C C/C A/A C/C C/C T/T A/A T/T C/C G/G C/T G/G A/G C/C C/C G/G C/C A/A C/C G/G T/T C/C G/G G/G A/A T/T C/C A/A A/A A/A T/T T/T C/C G/G A/A A/A A/A G/G T/T T/T T/T T/T T/T A/A T/T T/T A/A T/T A/A T/T C/C A/A G/G C/C A/A G/G A/G A/G C/C A/A A/A C/C G/G C/C A/A C/T C/C G/G C/C G/G A/A G/G A/A T/T A/A G/G C/C C/C C/C G/G G/G G/G G/G T/T C/C T/T T/T A/A A/A C/C A/G T/T A/A G/G G/G G/G G/G A/G T/T A/A A/A A/A T/T T/T T/T C/C G/G C/C G/G T/T T/T G/G C/C A/A T/T T/T C/C G/G A/A T/T A/G A/A G/G A/A T/T C/C G/G C/C C/C G/G C/C C/C T/T G/G T/T T/T A/A G/G G/G C/C C/C A/A T/T A/A T/T A/A G/G C/C C/C C/C T/T C/C C/C A/A G/G G/G G/G C/C C/C T/T G/G G/G A/A C/C T/T G/G A/A T/T T/T A/A A/A C/C T/T C/C C/C G/G A/A C/C C/C C/C G/G C/C T/T T/T G/G A/A T/T G/G A/A G/G T/T C/C A/C C/C A/A G/G G/G C/C C/C C/C T/T G/G A/A C/C C/C G/G A/A T/T C/C T/T G/G T/T A/A T/T C/C T/T T/T T/T G/G A/A C/C A/A A/A T/T T/T A/A A/A C/C A/A C/C G/G A/A C/C G/G C/C C/C T/T A/A C/C G/G T/T A/A A/A T/T C/C C/C C/C A/A C/C C/C C/C G/G A/A A/A C/C T/T G/G G/G T/T G/G A/A C/T C/C C/C C/C C/C T/T C/C C/C C/C A/A T/T C/C A/A C/C C/C A/A T/T G/G G/G C/C G/G C/C A/A T/T T/T T/T T/T A/A C/G A/A G/G T/T C/C A/A A/A T/T T/T A/A
2-203-4	Ceq6(CeqEP9)	Name: Ceq6; fingerprint code: T/T T/T A/G A/A A/A A/G C/T G/G A/G A/A C/C T/T C/C C/G T/T T/T C/T A/C A/G A/C G/G A/G T/T C/T A/A T/T A/G A/G T/T C/T G/G A/C T/T C/C A/G C/G C/T A/A A/A A/A C/C A/G A/G C/T C/T C/T C/T C/G C/G C/C G/T A/G A/G C/T C/T G/G G/G C/C A/G G/T C/C G/G C/C A/G A/A A/T A/G A/G G/T A/G G/G C/C G/G C/C G/T A/G A/G C/C A/T A/A C/T G/G G/G A/C T/T T/T T/T C/C T/T A/A T/T G/G A/G C/T C/T A/G A/C A/G C/T A/G A/T C/T A/G A/G A/G C/T C/T A/G A/G A/G C/T T/T T/T A/G A/C A/T A/C A/A T/T T/T T/T T/T T/T A/G C/C C/C A/C G/G A/A A/T C/T G/G A/G A/A G/G A/G A/G A/G A/C A/G A/G C/T T/T C/C C/C C/C T/T A/G T/T A/G A/A A/G A/G A/T A/C A/G C/T C/T C/T A/G A/G A/G T/T C/C T/T C/T C/C A/A A/A T/T A/G C/T A/G A/G C/G C/G G/G A/A C/C A/A A/A A/G C/T G/T C/T C/T A/A C/G A/G A/A C/T T/T C/T A/G C/C C/C A/C G/G A/A C/C A/A T/T T/T A/G A/T C/T C/G C/T C/T C/G C/T C/T A/T A/G C/T T/T A/T A/G C/G C/G C/T A/A C/C A/G A/A A/A A/G C/G C/G C/T C/T C/T A/C A/C A/G G/G A/G A/C C/G G/T A/G A/G A/G C/T A/A A/G A/T C/T G/G G/G A/T C/T C/T T/T T/T A/A G/G G/G T/T A/A T/T A/A C/C G/G C/C A/G C/T G/G A/G C/C C/C C/G C/C T/T A/A A/G A/G C/T C/T T/T C/C G/G A/A A/C C/T A/G A/G C/T A/C A/A A/G C/T G/G C/T C/T C/T C/T C/C A/A T/T T/T A/A G/G T/T C/C A/A G/G A/C A/G A/C A/G A/A C/T T/T A/A C/G G/T A/G T/T A/G T/T A/A A/G C/T C/T A/C C/T A/A C/C C/C C/C A/G A/T A/G C/G A/T A/A T/T C/C T/T G/G C/C T/T A/C C/T C/T A/T C/T C/T T/T A/G G/T C/T A/G C/T C/G A/T G/T G/G A/G C/T A/G C/T A/T T/T C/T C/T A/A A/A C/C A/A G/G A/T T/T G/G T/T C/T C/C A/T

1-211-3	Ceq51(CeqEP10)	Name: Ceq51; fingerprint code: A/A G/G A/G A/G A/G A/A C/T C/G A/A A/G C/T C/T C/T C/G A/A T/T C/T A/C A/G A/C A/A A/G T/T C/T A/G C/T G/G G/G C/T C/T A/G A/C C/T C/G A/G C/G C/T A/A A/G A/G C/C A/G G/G C/T C/T C/T C/C C/G C/G C/T G/T A/G G/G T/T C/C C/G A/A T/T G/G G/T C/T G/G C/G A/G A/G A/T A/G A/G G/T A/A A/G C/G A/G C/G G/G A/G A/G C/T A/T A/C C/T C/G A/G C/C C/T C/T A/T C/T C/C G/G T/T G/G A/G T/T C/C G/G C/C A/A C/C G/G T/T C/T A/G A/G A/G C/T C/T A/G A/G A/G C/T C/T C/T G/G A/A A/T A/A A/G T/T T/T C/C G/T T/T A/A T/T T/T A/A T/T A/G T/T C/C A/G A/G A/C A/G A/G A/G A/G A/A A/G A/G C/T T/T C/C C/C C/T T/T A/G C/T A/G A/A A/G A/G A/T A/C A/G C/T C/T C/T A/G A/G A/G G/T T/T C/C C/T C/T A/A A/A C/C A/A C/T A/G G/G C/G C/G A/A A/G T/T G/G T/T G/G C/C G/T C/T C/T G/G C/G A/G A/T C/C G/T C/T A/G C/T C/C A/C G/T G/G T/T A/G A/T G/G A/G A/T C/T C/G C/T C/T G/G C/C C/C A/T A/G C/T C/T A/T A/A C/C C/C C/T A/T C/T A/G A/T A/A G/G C/C C/G C/C C/C C/T A/C A/C A/G A/G G/G C/C C/C T/T A/G G/G A/G T/T A/T A/G A/T C/T G/T A/G A/T C/T C/T C/T C/T A/G A/G C/G C/T A/C G/T A/C C/T G/T C/G A/A C/T G/G A/A C/G C/T C/G C/C T/T A/A A/G A/G C/T C/T T/T C/T G/G C/C A/C C/T A/G A/G C/T C/C A/T A/G C/T G/G C/T C/T C/T C/T C/C A/G A/A C/C G/G A/G A/T T/T A/G G/G A/C A/G A/C G/G A/G C/T G/T A/C C/G G/T A/A C/T A/G T/T A/A A/G C/T C/T A/C C/C A/A C/C C/C C/C G/G A/A A/A C/C A/T A/A G/T C/T G/T A/G C/T C/T A/C C/T C/T A/T C/T C/T C/T A/G G/T C/T A/G C/T C/G T/T G/T G/T A/G C/T A/G C/C A/T C/T C/T C/T A/T A/C C/G A/G C/G T/T C/T A/A A/T T/T T/T A/T
2-437-2	Ceq7(CeqEP11)	Name: Ceq7; fingerprint code: A/A G/T A/G G/G A/A A/G C/T C/G A/A G/G C/C C/T C/T C/C T/T G/T C/T A/A G/G A/A A/A A/G C/T T/T G/G C/C A/A A/A C/C C/T G/G A/C T/T G/G G/G C/C T/T A/G A/A A/A A/C A/A A/G C/C C/C T/T C/C C/G C/C C/T T/T A/A A/G C/C T/T C/G G/G C/C A/A G/G C/T G/G C/G A/A A/A A/A A/G A/G G/T A/G A/A C/C G/G C/C T/T G/G G/G C/C T/T C/C T/T C/C A/G A/A T/T T/T T/T C/C C/C A/A T/T G/G A/A T/T T/T A/A A/A G/G T/T A/A A/A T/T A/A A/A A/A T/T T/T G/G G/G G/G C/C T/T T/T A/A C/C A/A C/C G/G C/C A/T C/C G/G A/T A/A C/C C/C C/C T/T A/G A/A T/T G/G A/A A/A G/G A/A G/G A/G A/C G/G G/G C/T T/T C/C C/C C/C T/T G/G T/T G/G G/G A/G G/G A/A C/C A/A T/T C/C C/C A/A A/A A/A T/T C/C T/T C/C C/T A/A G/G C/T A/A C/T A/G A/A C/C G/G A/G A/G C/C G/G A/T A/G C/C G/G C/C T/T G/G C/C A/G A/A T/T G/T T/T G/G C/C C/C A/A G/G A/A C/C A/A T/T T/T G/G A/A T/T C/C T/T T/T C/C T/T T/T A/A A/A C/C C/C A/A G/G C/G C/G T/T A/A C/C A/G A/A A/A A/G G/G G/G T/T C/C T/T A/A C/C A/A A/A A/G A/A G/G G/G G/G A/A A/A C/C T/T G/G T/T C/C G/G A/G A/T T/T C/C T/T T/T A/A G/G G/G T/T A/A T/T A/A C/T G/G C/C A/G C/T A/A G/G C/C C/C G/G A/A C/T A/G A/A A/A T/T T/T C/C C/T A/A C/C A/A T/T G/G G/G C/C A/A A/A A/A C/T A/G C/C T/T C/C T/T C/C A/A T/T T/T G/G A/G A/A T/T G/G G/G A/A G/G A/A G/G A/A T/T T/T A/A C/G G/T G/G C/T A/A T/T A/A A/G C/C T/T C/C T/T G/G T/T T/T T/T A/A T/T G/G G/G T/T A/A G/G T/T T/T A/A C/T T/T A/A T/T C/C A/A T/T T/T T/T G/G G/G T/T G/G T/T G/G T/T G/G T/T A/A T/T A/A C/C T/T T/T C/C C/C A/A C/C G/G G/G C/C A/A T/T G/G T/T C/C C/T T/T
1-447-3	Ceq8(CeqEP12)	Name: Ceq8; fingerprint code: A/A G/G A/G A/G G/G A/A T/T C/G A/G G/G C/T T/T T/T C/C T/T G/G C/T A/A G/G A/A A/A A/G C/T C/T G/G C/C A/A A/A C/C C/T G/G A/C C/T G/G G/G C/C T/T G/G A/A A/G A/A A/A A/G C/C C/C T/T C/T C/C C/C C/T T/T A/A A/A C/C C/T C/C G/G C/C A/A G/G C/T A/A G/G A/A A/A A/A A/G A/G T/T G/G G/G C/C A/A C/C T/T A/G A/G T/T T/T C/C C/T C/G A/G A/A T/T T/T T/T C/C C/C A/A T/T G/G A/A T/T T/T A/A A/A G/G T/T A/A A/A T/T A/A A/A G/G T/T T/T G/G G/G G/G C/C T/T T/T A/A C/C A/A C/C G/G C/C A/T C/C G/G A/A A/A C/C C/C C/C T/T A/G A/A T/T G/G A/A A/C A/G A/A G/G A/G A/C G/G G/G T/T T/T T/T A/A C/T C/C G/G C/T A/G G/G A/G G/G A/A C/C G/G T/T C/T C/C A/A A/G A/A G/G C/C T/T C/T C/T A/G G/G T/T G/G C/T A/A A/G C/G G/G A/A G/G C/C G/G A/T A/G C/C G/G C/T C/T G/G C/C A/G A/T T/T G/T T/T G/G C/C C/C A/A G/G A/A C/C A/A T/T T/T G/G A/A T/T C/C T/T T/T C/C T/T T/T A/A A/A C/C C/C T/T A/G G/G G/G C/T A/T C/T A/G A/T A/G A/A G/G G/G T/T C/C T/T A/A C/C A/A A/A A/G A/A G/G G/G G/G A/A A/A T/T T/T G/G T/T C/C G/G A/A T/T T/T C/C T/T T/T A/A G/G G/G T/T A/A T/T A/C T/T G/G C/C A/G C/T A/A G/G C/C C/C G/G A/A T/T A/G A/A A/A T/T T/T C/T C/C A/A C/C A/A T/T A/G G/G C/T A/A A/A A/A T/T A/A C/C T/T C/C C/T C/C A/A T/T T/T A/G G/G A/A C/C A/A A/A A/A G/G A/A G/G G/G C/T T/T A/A G/G G/G A/G C/T A/A A/T A/A A/G C/C T/T A/A T/T G/G T/T T/T T/T A/A T/T G/G G/G T/T A/A G/G C/C T/T A/A C/T T/T A/A T/T C/C A/A T/T T/T T/T G/G G/G T/T G/G T/T G/G T/T G/G T/T A/A T/T A/A C/T T/T T/T C/T C/T A/T C/C G/G G/G C/G A/A C/T G/G T/T C/C C/C T/T

3-225-1	CeqEP13	Name: CeqEP13; fingerprint code: A/T G/G A/G A/G A/G A/A C/T C/C G/G A/G T/T C/T C/T C/G A/T G/T C/T A/C A/G A/C A/G A/G T/T C/C A/G C/T G/G G/G C/T C/T A/G A/C C/T C/C A/A C/G C/T A/A A/G A/G A/A G/G G/G T/T C/T C/T C/C C/G C/G C/C G/T G/G A/A C/T C/T C/G A/G C/T A/G T/T C/T A/G C/G A/G A/G A/T A/G A/G G/G A/A A/G C/G A/G C/C G/T G/G A/A C/T A/T A/C C/C C/G A/G A/C C/T T/T A/T T/T C/C A/G C/T G/G A/G C/T C/T A/G C/C A/G C/T A/G A/T C/T A/G A/G A/G T/T C/C A/A A/A A/G C/T T/T C/T A/G A/C A/A A/C G/G C/T T/T C/T G/T A/T A/G T/T C/T A/C G/T A/A A/T C/C A/A G/G A/C A/G A/G A/G A/A A/C A/G A/G C/T G/T C/T A/C C/T C/T G/G C/T A/G A/A G/G A/G A/A A/C A/G C/T C/T C/T A/G A/G A/G G/T C/T C/T T/T C/T A/G A/G C/C A/G T/T A/A G/G G/G G/G A/G A/G C/T A/G T/T A/G C/T G/T C/T C/C A/G C/G A/G T/T C/T G/G C/C A/G C/T C/T A/C G/T A/G C/T A/G A/A G/T A/G A/T C/C C/G C/T C/T C/G C/T C/T A/T A/G C/T C/T A/T A/G C/G C/C C/C A/T T/T G/G A/T A/A G/G C/C C/G C/T C/T C/T C/C A/C A/G A/G G/G A/C C/G G/T G/G A/A A/A C/T A/T A/G A/T C/T G/T A/G A/T C/T C/T C/C C/T G/G A/G C/G C/T A/C G/T A/C C/T G/T C/G A/A C/T A/G A/G C/G C/T C/G A/C C/T A/G A/G A/G T/T C/T C/T T/T A/G A/C A/C C/T A/G A/G C/T C/C T/T G/G C/T A/G C/T C/T C/T C/T C/T A/G A/T C/T A/A A/G T/T C/T A/G A/G A/C A/G A/C A/G A/G C/T G/T A/C C/G G/T A/A C/T G/G T/T A/A A/A T/T C/C C/C C/T A/G C/T C/T C/T A/G A/T A/A C/G A/T A/G G/T C/T G/T A/G C/T C/T A/C C/T C/T T/T C/T C/C C/T A/A G/T C/T A/G C/T C/C A/T G/T G/T A/G C/T A/G C/T A/T C/T C/T C/T A/T A/C C/G A/G C/G T/T C/C A/A A/T C/T C/T A/T
2-189-4	CeqEP14	Name: CeqEP14; fingerprint code: A/T T/T G/G G/G G/G A/A C/C C/G A/A G/G T/T C/T T/T G/G A/A G/G T/T C/C A/A A/C G/G G/G T/T C/C A/A C/C G/G G/G T/T C/T A/A A/A C/C C/C G/G G/G C/C A/A G/G A/G A/C G/G G/G T/T T/T C/C T/T G/G G/G C/C G/G A/A G/G T/T C/C G/G A/A T/T G/G T/T C/C G/G C/C G/G G/G T/T G/G G/G G/G G/G G/G G/G G/G C/G T/T A/A G/G T/T A/T A/C C/T G/G A/A A/C C/C C/C A/A T/T C/C G/G C/C A/A G/G T/T C/C A/A C/C A/A C/C G/G T/T C/C G/G G/G A/A T/T T/T G/G G/G G/G T/T C/C C/C G/G A/A A/A A/A G/G T/T A/A C/C T/T A/A G/G C/C T/T A/C T/T G/G T/T T/T A/A G/G C/C A/A A/A A/G G/G A/C G/G A/A C/C G/G T/T C/C T/T T/T G/G C/C G/G A/A A/G G/G T/T C/C G/G C/C C/C C/C G/G G/G G/G G/G T/T C/C T/T C/C G/G A/A C/C G/G C/T A/A A/A G/G G/G G/G G/G C/C G/G T/T A/A T/T T/T T/T C/C G/G C/C G/G T/T T/T G/G C/C A/A C/C C/C C/C G/G G/G T/T G/G T/T G/G A/A T/T C/C G/G C/C C/T G/G C/C C/C T/T G/G T/T T/T A/A G/G G/G C/C C/T T/T T/T A/G T/T A/A G/G C/C C/C C/C C/C T/T C/C A/A G/G G/G G/G C/C C/C G/T G/G A/A A/A T/T A/A G/G T/T T/T T/T A/A A/A C/C T/T C/C C/C G/G A/A C/C C/C C/C T/T C/C T/T T/T G/G A/A C/C G/G A/G G/G C/T C/C C/C C/C A/A A/A G/G C/C C/C T/T T/T G/G C/C C/C C/C G/G G/G T/T C/C T/T G/G T/T G/G T/T C/C T/T T/T T/T A/G A/A C/C A/G A/A T/T T/T A/A A/G C/C A/A C/C A/A G/G C/T G/G C/C C/C T/T G/G C/C A/A A/A C/C A/A C/C C/T A/C C/T A/A T/T T/T T/T A/A A/A A/A C/C T/T A/G T/T T/T G/G A/G T/T T/T A/A C/C C/C A/A C/C C/C C/C A/A T/T C/C A/A C/C C/C A/A T/T T/T A/A C/C G/G C/C A/A T/T T/T T/T T/T A/A C/C G/G G/G A/A C/C G/G T/T T/T T/T T/T
6-207-1	Ceq52(CeqEP15)	Name: Ceq52; fingerprint code: T/T T/T A/A A/A G/G G/G T/T C/G A/A G/G C/T C/T C/T C/G A/T G/T C/T A/C A/G A/C A/G A/G C/C T/T A/G C/T A/G A/G C/T C/T A/G A/C C/C C/C A/G C/C C/T A/A A/G A/G A/C G/G A/G C/T T/T C/C T/T G/G C/C T/T T/T A/A A/G C/T C/C C/G A/G C/T A/G T/T C/C A/G C/G A/G A/G A/A A/A A/A G/T A/G A/G C/G A/A C/G G/G A/A A/G C/C A/A A/C C/T C/G A/G A/C C/T C/T T/T C/C C/T A/G C/T A/G A/G C/T C/T A/G A/C A/G C/T A/G A/T C/T G/G G/G A/A C/C C/T A/G A/G G/G C/T C/T C/T G/G A/A T/T A/A A/A T/T A/T T/T T/T T/T G/G C/T C/C A/C G/G A/A A/T C/T G/G A/G A/A G/G A/G A/G G/G A/C A/G A/G C/T G/T C/T A/C C/T C/T A/G T/T A/A A/G A/G A/G A/T A/C A/G C/T C/T C/T A/A A/A A/A G/T C/T C/T C/T C/T A/G A/G C/T A/A C/C G/G A/G C/G C/C A/G G/G C/T A/G A/A A/A C/C T/T C/T C/T A/G C/G A/G A/T C/C G/G C/C A/G C/T C/T A/C G/T G/G T/T G/G A/T G/T A/G A/T C/T C/G C/T C/T C/G C/T C/C T/T G/G T/T C/T A/T A/G C/C G/G C/T A/T C/T A/G A/A G/G A/A C/G C/G C/C T/T C/C A/C A/A A/G A/G A/G A/C C/G G/T A/G A/G A/G C/C A/T G/G A/T C/T G/G G/G T/T T/T C/C T/T T/T A/A G/G G/G T/T A/A T/T A/A C/C G/G C/C A/G C/T G/G G/G C/C C/C G/G C/C T/T A/G A/G A/A T/T T/T T/T C/T A/G A/C A/A C/T A/G A/G C/C C/C A/T A/A C/C G/G C/C T/T C/C C/C T/T G/G T/T T/T G/G G/G T/T C/C A/A A/A A/A G/G A/A A/A G/G T/T T/T A/A G/G G/G A/A T/T G/G T/T A/A A/G C/C T/T A/A T/T A/G C/T C/T C/T A/G A/T A/G C/G A/T A/G G/G C/C T/T A/A C/T T/T A/A T/T C/C A/T C/T C/T C/T A/G G/T C/T A/G C/T C/G A/T G/T G/T A/G C/T A/G C/C A/T C/C C/C C/C A/A C/C G/G G/G C/C A/T C/T A/G A/T T/T T/T A/T

3-270-1	Ceq9(CeqEP16)	Name: Ceq9; fingerprint code: A/A G/G A/G A/A A/G A/G T/T C/G A/A A/G C/T T/T T/T C/C A/T G/G C/T A/A G/G A/A A/A A/G C/C C/C G/G C/C A/A A/A C/C C/T G/G A/A C/T C/G G/G C/C T/T A/G A/A A/A A/A A/A G/G C/C C/C T/T C/T C/C C/C C/T T/T A/A A/A C/T C/T C/C G/G C/C A/A G/G C/T A/G C/C A/A A/A A/A A/G G/G T/T G/G G/G C/C G/G C/C T/T G/G G/G C/T A/T A/C C/T G/G A/A A/A T/T T/T T/T C/C T/T A/A T/T G/G A/A T/T T/T A/A A/A G/G T/T A/A A/A T/T A/G A/A G/G T/T T/T G/G G/G G/G C/C T/T T/T A/A C/C A/A C/C G/G C/C A/T C/C G/G T/T A/A C/T C/C C/C G/T A/G T/T C/C G/G A/A A/C G/G A/A G/G A/G A/C G/G G/G C/C G/G C/C A/A C/C C/T A/A T/T A/A A/G A/A G/G A/A C/C A/A T/T T/T T/T A/A G/G A/A G/G C/C T/T C/C C/C A/A G/G T/T G/G C/T A/A A/A G/G C/C A/G A/G C/C G/G T/T G/G C/C T/T C/C T/T G/G C/C A/G A/T T/T G/T T/T G/G C/C C/C A/A G/G A/G C/C A/A T/T T/T G/G A/A T/T C/C T/T T/T C/C T/T T/T A/A A/A C/C C/T A/T A/G C/G C/G C/T A/T C/T G/G T/T A/G A/A G/G G/G T/T C/C T/T A/A C/C A/A A/G G/G A/A G/G G/G G/G A/A A/A T/T T/T G/G T/T C/C G/G G/G A/T T/T C/C T/T T/T G/G G/G G/G T/T A/A T/T A/A T/T G/G C/C A/G C/T A/G G/G C/C C/C G/G A/A C/C A/G A/A A/A T/T T/T C/T C/C A/A C/C A/A T/T A/G A/G C/T A/A A/A A/A C/C G/G C/C T/T C/C C/C C/C A/A T/T T/T G/G A/A A/A C/T A/G A/A A/A G/G A/A G/G A/G C/T T/T A/A G/G G/G G/G T/T A/G A/A A/A G/G C/C C/C C/C T/T G/G C/T T/T C/T A/A T/T G/G G/G T/T A/G G/G T/T T/T A/G C/T T/T A/A T/T C/C A/A T/T T/T T/T G/G G/G T/T G/G T/T G/G T/T G/G T/T A/A T/T A/A C/T T/T C/C T/T T/T A/T C/C G/G G/G C/C A/A T/T A/G T/T C/C C/T A/T
6-195-3	CeqEP17	Name: CeqEP17; fingerprint code: A/A G/T A/G A/G A/G A/G T/T C/G A/A A/A C/C T/T C/T C/G T/T T/T C/T A/C A/G A/C A/G A/G T/T C/T A/G C/T G/G G/G T/T T/T A/G A/C C/C C/C A/G C/C C/T A/A A/G A/G A/C A/G A/G C/T C/T C/T C/T C/G C/C T/T T/T A/G A/G C/T C/T C/G G/G C/C A/A G/T C/C A/G C/C A/G A/G A/T A/G A/G T/T A/A A/A C/C A/G G/G G/G A/A A/A C/C A/A A/C C/T C/G A/G A/A C/T C/T A/A T/T C/T G/G C/C A/A A/G C/T C/C A/A A/A G/G C/T A/A A/A C/T A/G A/A A/G C/T C/C A/A A/A A/A T/T C/C C/C G/G A/A T/T A/A A/A T/T A/T C/T G/T T/T G/G C/T C/C A/C G/T A/A T/T T/T A/G A/G A/A G/G A/A A/G A/G A/C G/G G/G T/T T/T C/T C/C C/C C/T A/G C/T A/A A/G A/G A/A A/T A/C A/G C/T C/T C/T A/G A/G A/G G/G T/T C/C T/T C/C G/G A/G C/T A/G C/T A/G A/G C/C C/G A/G A/A C/C A/G A/T A/G C/T G/T C/T C/T A/A C/G A/G A/A C/T G/T C/T A/A C/T C/T C/C G/T G/G C/T A/G A/T G/T G/G A/A T/T C/C T/T T/T C/C T/T T/T A/A A/A C/C T/T A/T A/A C/C C/G C/T A/A C/C A/A A/A G/G A/A C/G C/G C/T C/T C/T C/C A/A G/G G/G A/A C/C C/C T/T A/A G/G A/G C/C T/T A/A A/T C/T G/T A/G A/T C/T C/T C/T C/T A/G A/G C/G C/T C/C G/T A/C C/T G/T C/G G/G C/T G/G A/G C/G C/T G/G A/C C/T G/G G/G A/G C/T C/T T/T C/T A/G A/C A/A T/T A/A A/G C/C C/C A/A A/A C/C G/G C/T T/T C/C C/T C/T A/G A/T C/T A/G G/G A/T C/T A/G G/G A/A A/G A/C A/G A/G T/T G/T A/A C/G G/T A/G C/T G/G A/T A/C A/G C/T C/T A/A C/T A/G C/T C/T C/T A/G A/T A/G C/G T/T A/G G/T C/T G/T A/G C/T C/T A/C C/T C/C A/A T/T C/T C/T A/G G/T C/T A/G C/T C/G A/T G/T T/T A/G C/T A/G T/T A/T C/T T/T T/T A/A A/C C/G A/G C/G A/T C/C G/G A/T T/T T/T T/T
6-71-3	Ceq10(CeqEP18)	Name: Ceq10; fingerprint code: T/T G/T A/G A/A A/G A/G T/T C/G A/A A/A C/C T/T C/C C/C T/T T/T C/C A/C A/G A/A A/A A/A T/T C/C A/G C/T A/G A/G C/T C/T A/G A/A T/T C/G G/G C/G C/T A/G A/A A/A A/C A/A A/G C/T C/T C/T C/T C/G C/G T/T T/T G/G G/G C/T T/T G/G A/G C/T A/G G/T C/C A/A C/G A/A A/A A/T A/G A/G T/T A/A A/A C/C A/G C/G G/T G/G G/G C/C A/A A/A C/T C/G A/G A/C C/T C/T A/A C/T T/T A/A C/T A/G A/G C/C T/T A/G A/C A/G C/T A/G A/T C/C A/G A/G A/A T/T T/T G/G G/G G/G C/T C/T C/T A/G A/C A/T A/C G/G C/C A/T C/C G/G A/T A/G C/C C/T C/C G/G A/A A/A C/C A/G A/A A/A G/G G/G A/A A/G A/A A/G A/A C/T G/T C/C A/C C/T C/T G/G T/T A/A A/G A/A A/G A/T A/C A/A T/T T/T T/T A/A A/G A/A G/T C/T C/T T/T C/T G/G A/A T/T A/A C/C G/G A/G C/G C/G A/G G/G C/T G/G T/T G/G C/C G/G C/C T/T A/G C/G A/A A/A C/T G/T C/T A/A C/T T/T C/C G/T A/G C/T A/A A/T G/T A/G A/T C/T C/G C/T C/T C/G C/T C/T A/T A/G C/T C/T T/T A/G C/C C/C C/T A/T C/T A/G A/A A/G A/G G/G C/G C/T C/T C/T A/C A/C A/A A/A G/G A/A G/G G/G A/G A/G G/G C/T T/T A/G A/T C/T G/T A/G A/T C/T C/C T/T T/T G/G G/G G/G C/T A/C G/T A/A C/T G/T C/G A/G C/C A/G G/G C/G C/T C/G C/C C/C G/G A/A A/A C/T T/T C/T C/T G/G A/A A/A C/T A/G A/G C/C C/C A/T A/G C/C G/G C/T C/T C/T C/T C/T A/G A/T C/T G/G G/G A/A T/T A/A A/G A/C G/G A/A A/G A/G C/T G/T A/A G/G G/G A/G C/C A/G A/T C/C A/G C/C T/T A/A C/T A/G C/T C/T C/T G/G A/A A/A C/C A/A A/G G/T C/C T/T G/G C/C T/T A/A T/T T/T A/T T/T T/T T/T G/G G/G T/T G/G T/T G/G T/T G/G T/T A/A T/T A/A T/T T/T C/T C/T T/T A/A C/C G/G G/G C/C A/T C/C G/G T/T C/T C/T A/T

6-147-3	Ceq11(CeqEP19)	Name: Ceq11; fingerprint code: T/T T/T A/A A/A G/G G/G C/C C/G G/G G/G T/T C/T T/T G/G A/A T/T T/T C/C A/A C/C G/G G/G T/T C/C A/A T/T G/G G/G T/T T/T A/A A/A C/C C/C A/A G/G C/C A/G A/A A/A C/C G/G A/A T/T T/T C/C T/T G/G G/G C/C G/G A/A G/G T/T C/C G/G A/A T/T G/G T/T T/T G/G C/C G/G G/G T/T G/G G/G G/G G/G G/G G/G G/G G/G G/G A/A A/A C/C A/A A/A C/C G/G G/G C/C C/C C/C A/A T/T C/C G/G C/C A/A A/G C/C C/C G/G C/C A/A C/C G/G T/T C/C G/G G/G A/A C/C C/C A/A A/A A/A T/T C/C C/C G/G A/A T/T A/A A/A T/T T/T T/T T/T A/A G/G T/T T/T A/A T/T G/G T/T T/T A/A G/G C/C A/A G/G A/A G/G A/C A/A A/A C/C G/G T/T C/C T/T T/T A/A C/C G/G A/A A/G A/A T/T A/A G/G C/C C/C C/C G/G G/G G/G G/G T/T C/C T/T C/C G/G G/G C/C G/G T/T A/A A/A G/G G/G G/G A/A C/C A/A A/A A/A T/T T/T T/T C/C A/A G/G A/A T/T C/C G/G C/C A/A T/T T/T C/C T/T G/G T/T G/G A/A G/G A/A T/T C/C G/G C/C C/C G/G C/C C/C T/T G/G T/T T/T A/A A/A C/C C/C C/C T/T T/T G/G A/A A/A G/G C/C C/C C/C T/T C/C C/C A/A G/G G/G A/A C/C C/C T/T A/A G/G G/G T/T A/A A/A A/A T/T T/T A/A A/A C/C T/T C/C C/C G/G A/A C/C C/C C/C G/G C/C T/T T/T G/G A/A C/C A/A A/A G/G T/T C/C C/C C/C A/A G/G G/G C/C C/C T/T T/T G/G C/C C/C C/C G/G G/G T/T A/A T/T G/G T/T A/A T/T C/C T/T T/T T/T G/G A/A C/C A/A A/A T/T C/C G/G G/G C/C A/A C/C A/A G/G T/T G/G C/C C/C T/T G/G C/C A/A A/A C/C A/A T/T C/C C/C C/C A/A C/C C/C C/C G/G A/A A/A C/C T/T A/G T/T T/T G/G G/G C/C C/C C/C C/C C/C T/T C/C C/C C/C A/A T/T C/C A/A C/C C/C A/A T/T G/G G/G C/C G/G C/C A/A T/T T/T T/T A/A A/A C/C A/A G/G A/A C/C A/A A/A T/T T/T T/T
4-440-2	Ceq53(CeqEP20)	Name: Ceq53; fingerprint code: A/A G/T A/G A/G A/A A/A C/T C/G A/G A/G C/T T/T T/T C/C T/T G/G C/T A/A G/G A/A A/A A/G C/C T/T G/G C/C A/A A/A C/C C/T G/G A/C C/T C/G A/G C/C T/T G/G A/A A/A A/A A/A A/A C/C C/C T/T C/T C/C C/C C/T T/T A/A A/A C/C T/T C/C G/G C/C A/A G/G C/C A/A C/G A/A A/G A/A A/A A/A T/T G/G G/G C/C G/G C/C T/T A/G G/G C/C T/T C/C T/T C/C A/A A/A T/T T/T T/T C/C C/C A/A T/T A/G A/A T/T T/T A/A A/A G/G T/T A/A A/A T/T A/A A/A G/G T/T T/T G/G G/G G/G C/C T/T T/T A/A C/C A/A C/C G/G C/C A/T C/C G/G A/T A/A C/T C/C C/C G/T A/A T/T C/C G/G A/A A/A G/G A/A G/G A/G A/C A/G G/G C/T G/T C/T A/C C/C C/C A/G T/T A/A A/G A/A G/G A/A C/C G/G T/T C/C C/T A/A G/G A/A T/T C/C T/T C/C C/C A/A G/G C/T A/G C/T A/G A/A C/G G/G A/A A/G C/C G/G T/T A/A C/C G/G T/T C/C G/G C/C A/G T/T T/T G/T T/T G/G C/C C/C A/A G/G G/G C/T A/A T/T T/T G/G A/A T/T C/C T/T T/T C/C T/T T/T A/A A/A C/C C/C A/T A/G C/C C/G C/T A/T C/T A/G A/T A/G A/A G/G G/G T/T C/C T/T A/A C/C A/A A/A A/A A/A G/G G/G G/G A/A A/A C/T T/T G/G T/T C/C G/G A/G T/T T/T C/C T/T T/T A/G G/G G/G T/T A/A T/T A/C C/T G/G C/C A/G C/T A/A G/G C/C C/C G/G A/A T/T A/G A/A A/A T/T T/T C/C C/T A/A C/C A/A T/T A/G G/G T/T A/C A/A A/A C/T A/G C/C T/T C/C T/T C/C A/A T/T T/T A/A G/G A/T C/C G/G A/G A/A G/G A/A G/G A/G C/T T/T A/C G/G G/G G/G T/T A/G A/T A/C A/G C/C C/T A/C T/T G/G T/T T/T T/T A/A T/T G/G G/G A/T A/G G/G C/C T/T A/A C/T T/T A/A T/T C/C A/A T/T T/T T/T G/G G/G T/T G/G T/T G/G T/T G/G T/T A/A T/T A/A C/T T/T C/C C/T C/T A/T C/C G/G G/G C/G A/A C/T A/G T/T C/C C/C T/T
1-216-2	Ceq12(CeqEP21)	Name: Ceq12; fingerprint code: A/T T/T A/A A/A G/G G/G C/C C/G A/G G/G T/T C/T C/T C/G A/T G/T C/T A/A G/G A/A A/G A/G C/T C/T A/G T/T A/A A/A C/C C/T G/G A/C T/T C/G G/G C/G C/C A/G A/G A/A A/C G/G A/A T/T T/T C/C T/T G/G C/C C/T T/T G/G G/G T/T C/C G/G G/G C/C A/A G/G C/C A/G C/C A/G A/G A/T A/G A/G G/G A/G A/A C/C A/A G/G G/T A/G A/A C/C A/A A/A T/T C/G A/G A/C C/T C/T A/T C/T T/T A/A C/C A/A A/G C/T C/T A/A A/C A/G C/C A/G A/T C/T A/G A/G A/G C/C C/T A/G A/G A/G C/C C/T T/T A/G A/C A/T A/C A/G C/T A/T C/T G/T T/T A/A T/T C/T A/C G/T A/G T/T C/C G/G A/A C/C A/G A/A G/G A/G A/C A/G A/G C/T G/T C/C A/C C/T C/T A/G T/T A/A A/G A/G G/G A/T A/C G/G C/C C/C C/C A/A G/G A/A T/T C/T C/T C/T C/C A/A G/G T/T A/G C/C G/G G/G C/G C/C A/G A/G C/T A/G A/T A/G C/T T/T T/T C/C A/G C/C A/G A/T C/T G/T C/T A/G C/C C/T A/C G/T G/G T/T G/G A/T G/T A/G A/T C/T C/G C/T C/T C/G C/T C/T A/T A/G C/T T/T T/T A/G C/C C/G C/T A/A C/C A/A A/T G/G A/A G/G C/G C/T C/T C/T A/C A/C G/G G/G A/A C/C C/C T/T A/A A/G G/G C/T A/T G/G A/A T/T G/T A/G T/T T/T C/C T/T T/T A/A G/G G/G T/T A/A T/T A/A C/C G/T C/G A/G C/T A/G A/G C/C C/C G/G A/C T/T A/A A/G A/G C/T T/T T/T C/T A/G A/C A/A C/T A/G A/G C/T A/C A/T A/G C/C G/G C/C T/T C/C C/C C/T A/G T/T T/T G/G A/G A/A T/T A/G G/G A/C A/G A/C A/G G/G T/T T/T C/C C/C T/T G/G C/T A/A A/A A/C A/A C/T C/T A/C C/T A/G C/T C/T C/T A/G A/T A/G C/C A/T A/G T/T C/T G/G A/G C/C C/T A/C C/T C/T A/T C/T C/T C/T A/G G/T C/T A/G C/T C/G A/T G/T G/T A/G C/T A/G C/T A/T C/T C/C C/C A/A C/C C/G A/G C/G A/T C/T G/G T/T C/T C/T A/T

6-394-4	Ceq13(CeqEP22)	Name: Ceq13; fingerprint code: T/T T/T A/A A/A G/G G/G C/C C/G A/G A/A C/T C/T C/T C/G A/T T/T C/T A/C A/G A/C A/G A/G C/T T/T A/G C/T A/G A/G C/T C/T A/G A/C C/T C/C G/G C/C T/T G/G G/G A/G C/C A/A G/G C/C C/T C/C T/T G/G C/G T/T G/T A/G A/G C/T C/C C/C A/G C/T A/G G/T C/C A/G C/C A/A A/G A/A A/G A/G G/T A/G A/A C/C A/G C/C G/T A/G G/G C/C A/A A/A C/T G/G G/G A/C C/C C/C A/A T/T C/T A/A T/T G/G A/G C/T C/T A/G A/C G/G C/T A/G A/T C/T A/G A/A A/G C/T C/C G/G A/G A/G C/T C/T C/T A/G A/C T/T A/A A/G C/T T/T C/T G/T T/T A/G C/C C/T C/C G/G A/G A/T C/T A/G A/G A/C A/G G/G A/A G/G A/A A/A A/A C/T G/T C/T A/C C/T T/T G/G C/T A/A A/A A/G A/G A/T A/C A/G T/T T/T T/T A/A A/A A/A T/T C/T C/T C/T T/T A/G A/G C/T A/A C/T A/G A/G C/G C/G A/A A/G C/T A/A A/T G/G C/C G/T C/T C/T G/G G/G A/G A/T C/T G/T C/T A/G C/T C/T A/C T/T A/G C/T A/G A/T G/T A/G A/T C/T C/G C/T C/T C/G C/T C/T A/A A/A C/C C/T T/T A/G C/G C/C C/T A/T C/T A/G A/A A/G G/G C/C G/G C/T C/T C/T A/C A/C A/A A/A A/G A/C C/G G/T A/G A/G A/G C/C T/T G/G A/T C/T G/T A/A A/T C/T C/T C/T C/T A/G A/G G/G C/T A/C G/T A/A C/C G/T C/G G/G C/T G/G A/G C/C C/C C/G A/C C/T G/G A/G G/G C/C C/C T/T T/T G/G C/C A/C T/T A/G A/A C/C C/C A/T A/G C/T G/G C/T C/T C/T C/T C/C A/A A/T C/T G/G A/G A/T C/C A/G G/G A/A A/A C/C G/G A/A T/T T/T A/A C/G G/T A/G C/T A/G A/T A/C A/A C/T C/T A/A T/T A/G T/T C/T C/T A/G A/T A/G C/G A/T A/G T/T C/T G/T G/G C/C C/T A/A T/T C/T A/T T/T T/T T/T G/G G/G T/T G/G T/T G/G T/T G/G T/T A/A T/T A/A C/T T/T C/C C/T C/T A/A C/C G/G G/G C/C A/T C/T A/G A/A C/C C/C A/T
6-283-2	Ceq14(CeqEP23)	Name: Ceq14; fingerprint code: A/A G/T A/G A/G A/A A/A C/T G/G A/G G/G C/C C/T T/T C/C A/T T/T C/T A/A G/G A/A A/A A/G T/T T/T G/G C/C A/A A/A C/C C/T G/G A/C C/T C/G G/G C/C T/T G/G G/G G/G C/C A/G A/A C/C C/C T/T C/C C/C C/C C/T T/T A/G G/G C/C C/T C/G G/G C/C A/A G/T C/C A/A G/G A/A A/G A/A G/G G/G T/T G/G A/G C/G G/G C/C T/T G/G G/G C/T T/T C/C C/T G/G A/A C/C T/T T/T T/T C/C C/T A/A T/T A/G A/A T/T T/T A/A A/A G/G T/T A/A A/A T/T A/A A/A G/G T/T T/T G/G G/G G/G C/C T/T T/T A/A C/C A/A C/C G/G C/C A/T C/C G/G A/T A/A C/C C/C C/C G/T A/G A/T T/T G/G A/A C/C A/G A/A A/G A/G A/C G/G G/G T/T T/T C/T C/C C/T C/T A/G C/T G/G G/G G/G G/G A/A C/C A/G T/T C/T C/T A/A A/A A/A G/G C/C T/T C/C C/C A/A G/G C/T A/A C/C G/G A/A G/G C/G A/A G/G C/C G/G A/T A/A C/C G/G C/C T/T G/G C/C A/G A/A T/T G/T T/T G/G C/C C/C A/A G/G A/A C/C A/A T/T T/T G/G A/A T/T C/C T/T T/T C/C T/T T/T A/A A/A C/C C/C T/T A/A G/G G/G C/C A/T T/T A/G T/T G/G A/A G/G G/G T/T C/C T/T A/A C/C A/A A/A A/G A/A G/G G/G G/G A/A A/A C/T T/T G/G T/T C/C G/G G/G A/A T/T C/C T/T T/T A/G G/G G/G T/T A/A T/T A/A T/T G/G C/C A/G C/T A/A G/G C/C C/C G/G A/A C/T A/A A/A A/A T/T T/T C/T T/T A/A C/C A/A T/T G/G G/G C/T A/C A/A A/A C/T A/G C/C T/T C/C T/T C/C A/A T/T T/T A/G G/G A/A C/T A/G A/A A/A G/G A/A G/G A/G C/T T/T A/C G/G G/T G/G T/T A/A A/A A/C G/G C/C C/C C/C T/T G/G T/T T/T T/T A/A T/T G/G G/G A/A A/G G/G C/T T/T A/A C/T T/T A/A T/T C/T A/A T/T T/T T/T G/G G/G T/T G/G T/T G/G T/T G/G T/T A/A T/T A/A C/T T/T T/T C/C C/C A/A C/C G/G G/G C/C A/A T/T G/G T/T C/T C/C A/T
3-80-2	Ceq54(CeqEP24)	Name: Ceq54; fingerprint code: A/A T/T A/G A/G A/G A/G C/T C/G A/A G/G C/T T/T C/C C/C A/T T/T C/T A/A G/G A/A A/A A/G T/T C/C A/G C/T A/G A/G C/T C/T G/G A/C T/T C/G A/G C/G C/T A/A A/G A/G C/C G/G A/G C/T C/T C/T C/T C/G C/G C/T G/T A/G A/G C/C T/T G/G A/G C/T A/A G/T C/C A/A G/G A/A A/A A/A A/A A/A T/T A/G A/A C/G A/A C/G G/G A/A A/G C/C A/T A/C C/T G/G A/G C/C C/C C/T A/T C/T C/T A/G T/T G/G A/G C/C C/C G/G A/C A/G C/T A/G A/T C/T A/G A/G A/G T/T C/T A/G A/G A/G C/T C/T C/T A/G A/C A/T A/C A/G C/T A/T C/T G/T T/T A/G C/C C/C A/C G/G A/A A/A C/T A/G A/A A/C G/G A/G A/A G/G A/A A/A A/A C/T G/T C/C A/C T/T T/T A/G T/T A/A A/G A/G A/G A/T A/C A/A C/T T/T T/T A/G A/G A/G G/T C/T C/C C/T C/C A/G A/G T/T A/G C/T A/G G/G C/C C/C G/G G/G T/T A/G A/A A/A T/T T/T T/T C/C A/A C/G A/A A/A T/T G/T T/T A/G C/T C/T A/C G/T A/G C/T A/G A/T G/T A/G A/T C/T C/G C/T C/T C/G C/T C/T A/T A/G C/T T/T A/T G/G C/C C/G C/C T/T T/T G/G A/A A/G A/A G/G G/G C/T C/C T/T A/A C/C A/G A/G G/G A/C C/G G/T A/G A/G A/G C/T A/T A/A T/T C/C G/G G/G A/T C/T C/T C/T C/T A/G A/G C/G C/T A/C G/T A/A C/C G/G C/C A/A C/C A/G G/G C/C C/C C/G A/C C/T A/G A/A A/G C/T C/T T/T T/T A/A C/C A/A C/T A/G G/G C/C A/C A/T A/G C/T G/G C/C T/T C/C C/C C/T A/G T/T C/T A/G G/G A/T C/C A/A A/G A/C A/A A/C G/G A/A C/T T/T A/A G/G G/G G/G T/T A/A T/T A/A A/A C/T T/T A/A T/T G/G T/T T/T C/T A/G A/T A/G C/G A/A A/G G/T C/T G/G G/G C/C C/T A/C C/T C/T A/T T/T T/T T/T G/G G/T T/T G/G T/T G/G T/T G/G T/T A/A T/T A/A C/T T/T C/T C/C C/C A/A A/C G/G A/G C/C A/T C/T G/G T/T T/T T/T A/T

6-388-2	Ceq15(CeqEP25)	Name: Ceq15; fingerprint code: T/T T/T A/A A/A G/G A/G C/C C/C G/G G/G T/T C/T T/T G/G A/A G/G T/T C/C A/A A/C A/G G/G T/T C/C A/A T/T G/G G/G T/T C/T A/A A/C C/C C/C A/A G/G C/C A/A A/A A/A A/C G/G A/A T/T T/T C/C C/T G/G G/G C/C G/G A/A G/G T/T C/C G/G A/A T/T G/G T/T T/T G/G C/C G/G G/G T/T G/G G/G G/G G/G G/G G/G G/G G/G G/G A/G A/A T/T A/T A/C C/T G/G G/G C/C C/C C/C A/A T/T C/C A/G C/C A/A A/G C/C C/C G/G C/C A/A C/C G/G T/T C/C G/G G/G A/A C/C C/C A/G A/A A/A T/T C/C C/C G/G A/A T/T A/A A/A T/T A/T T/T T/T A/T G/G T/T T/T A/A T/T G/G T/T T/T A/A G/G C/C A/A A/G G/G G/G A/C A/G G/G C/C G/G T/T C/C C/T T/T A/A C/C G/G A/A A/G A/A T/T A/A G/G C/C C/C C/C G/G G/G G/G G/G T/T C/C T/T T/T A/A G/G C/C G/G T/T A/A G/G G/G G/G G/G A/G C/T A/G A/A A/G T/T T/T T/T C/C A/A G/G A/A T/T C/T G/G C/C A/A T/T T/T C/C T/T G/G T/T G/G A/A G/G A/A T/T C/C G/G C/C C/C G/G C/C C/C T/T G/G T/T T/T A/T A/A C/G C/C C/C T/T T/T A/G A/T A/A G/G C/C C/C C/C T/T C/C C/C A/A G/G G/G A/A C/C C/C T/T A/A G/G G/G T/T A/A A/A A/A T/T T/T A/A A/A C/C T/T C/C C/C G/G A/A C/C C/C C/C G/G C/C T/T T/T G/G A/A C/C G/G A/A G/G T/T C/C C/C C/C G/G G/G G/G C/C C/C T/T T/T G/G C/C C/C C/C G/G G/G T/T A/A T/T A/G C/T A/G T/T C/C T/T T/T T/T G/G A/A C/C A/A A/A T/T C/C A/G G/G C/C A/A C/C A/A G/G T/T G/G C/C C/C T/T G/G C/C A/A A/A C/C A/A T/T C/C C/C C/C A/A C/T C/C C/C G/G A/A A/A C/C T/T A/G G/T T/T G/G G/G C/C C/C C/C C/C T/T T/T C/C C/C C/C A/A T/T C/C A/A C/C C/C A/A T/T G/G G/G C/C G/G C/C A/A T/T T/T T/T T/T A/C C/C A/A G/G A/A C/C A/A A/A T/T T/T T/T
6-201-3	Ceq16(CeqEP26)	Name: Ceq16; fingerprint code: A/A G/G A/A A/A G/G A/A C/C C/C G/G A/A T/T C/T T/T G/G A/A G/T C/C C/C A/A C/C A/A G/G T/T C/C A/A T/T G/G G/G C/C C/T A/A C/C C/C C/C A/A G/G C/C A/A G/G G/G A/A G/G G/G T/T T/T C/C C/C G/G G/G C/C G/G G/G G/G T/T C/C C/C A/A T/T G/G T/T T/T G/G C/C G/G G/G T/T G/G G/G G/G A/A G/G G/G G/G C/C G/G G/G A/A T/T T/T C/C C/C C/C A/A C/C C/C T/T A/T T/T C/C G/G C/C G/G A/G C/C C/C G/G C/C A/A C/C G/G T/T C/C G/G G/G A/A T/T C/C A/A A/A A/A T/T T/T C/C G/G A/A A/A A/A G/G T/T T/T C/T T/T A/A A/A T/T T/T A/A T/T A/A T/T C/C A/A G/G C/C A/A G/G A/G A/G C/C A/A A/A C/C G/G C/C A/A C/T C/T G/G C/C G/G A/A G/G A/A T/T A/A G/G C/C C/C C/C G/G G/G G/G G/G T/T C/C T/T T/T A/A A/A C/T A/G T/T A/A G/G G/G G/G G/G A/G T/T A/A A/A A/A T/T T/T T/T C/C G/G C/C G/G T/T T/T G/G C/C A/A T/T T/T C/C G/G A/A T/T A/G A/A G/G A/A T/T C/C G/G C/C C/C G/G C/C C/C T/T G/G T/T T/T A/A G/G G/G C/C C/C A/A T/T A/G T/T A/A G/G C/C C/C C/C T/T C/C C/C A/A G/G G/G G/G C/C C/C T/T G/G G/G A/A C/C T/T G/G A/A T/T T/T A/A A/A C/C T/T C/C C/C G/G A/A C/C C/C C/C G/G C/C T/T T/T G/G A/A T/T G/G A/A G/G T/T C/C A/C C/C A/A G/G G/G C/C C/C C/C T/T G/G A/A C/C C/C G/G A/A T/T C/C T/T G/G T/T A/A T/T C/C T/T T/T T/T G/G A/A C/C A/A A/A T/T T/T A/G A/A C/C A/A C/C G/G A/A C/C G/G C/C C/C T/T A/A C/C G/G T/T A/A A/A T/T C/C C/C C/C A/A C/C C/C C/C G/G A/A A/A C/C T/T G/G G/G T/T G/G A/A T/T C/C C/C C/C C/C A/T C/C C/C C/C A/A T/T C/C A/A C/C C/C A/A T/T G/G G/G C/C G/G C/C A/A T/T T/T T/T T/T A/A C/C A/A G/G T/T C/C A/A A/A T/T T/T A/A
3-397-1	Ceq17(CeqEP27)	Name: Ceq17; fingerprint code: A/A G/G G/G G/G A/A A/A T/T G/G A/A G/G T/T C/T T/T G/G T/T T/T C/T C/C A/A C/C G/G A/G C/C T/T A/G C/T A/G A/G C/T C/T G/G A/C C/C C/C G/G C/G C/C A/G A/G A/G C/C A/G G/G C/C C/C T/T C/C C/C C/C T/T T/T G/G G/G C/T C/T G/G G/G C/C A/A T/T C/T A/A G/G A/A A/A A/A A/A A/A G/T A/G A/G C/C A/G C/G G/T A/A A/G C/C A/T A/A C/T C/G G/G A/C C/C C/C A/T C/T T/T A/G C/T A/G A/G C/T C/T A/G A/A A/G C/T A/G A/T C/T G/G A/G A/G C/T C/T A/G A/G A/G C/T C/T T/T A/A C/C A/A A/C A/G C/T A/T C/T T/T A/T G/G T/T C/C C/C G/G A/A A/A C/T A/G A/G A/C G/G A/G A/G G/G A/C A/G A/G T/T T/T C/C A/C T/T C/T A/G C/T A/A G/G A/A A/G A/T A/C A/G C/C C/T C/T A/G A/G A/A G/T T/T C/T C/T C/T G/G G/G C/T A/A C/C G/G G/G C/G C/C A/G G/G C/T G/G T/T A/G C/C G/T C/T C/T A/G G/G A/G T/T C/C T/T C/C A/A C/T C/T C/C T/T G/G C/C A/A T/T T/T G/G A/A T/T C/C T/T T/T C/C T/T T/T A/A A/A C/C C/T T/T A/A C/G C/G C/T A/A C/C A/G A/A A/G A/G C/G C/C C/C C/T C/T A/C A/C A/G G/G A/G A/C C/G G/T A/G A/G A/A C/C A/T A/G A/T C/T G/G A/G A/T C/T C/T C/T C/T G/G A/G C/G C/T A/C G/T A/A C/C G/T C/G G/G C/T A/G A/G G/G T/T C/C C/C C/C A/A G/G A/G C/T C/T T/T C/T G/G A/A A/A T/T A/G G/G C/C C/C A/A A/A C/C G/G C/C T/T C/C C/C C/C A/A A/T C/T A/G A/A T/T C/C G/G G/G A/A A/G A/C G/G A/G C/T T/T A/A C/C T/T G/G C/C A/A A/A C/C A/A T/T C/C A/A T/T G/G T/T T/T C/T A/A A/T A/G C/G A/A A/G G/G C/T T/T G/G C/T C/T A/C C/T C/C A/T C/T C/T C/T A/G G/T C/T G/G T/T G/G T/T G/G T/T A/A T/T A/A C/T T/T C/C C/T C/C A/A A/C C/G G/G C/C A/T C/T A/G A/T C/T C/T A/T

2-286-2	Ceq18(CeqEP28)		Name: Ceq18; fingerprint code: T/T T/T A/G A/A G/G A/G C/C C/G G/G A/G T/T C/T T/T G/G A/A G/G T/T C/C A/A C/C G/G G/G T/T C/C A/A T/T G/G G/G T/T C/T A/A A/A C/C C/C A/A G/G C/C A/A A/A A/A C/C G/G A/A T/T T/T C/C C/C G/G G/G C/C G/G A/A G/G T/T C/C G/G A/A T/T G/G T/T T/T G/G C/C G/G G/G T/T G/G G/G G/G G/G G/G G/G A/G G/G G/G A/A A/A T/T A/T A/C C/T G/G G/G C/C C/C C/C A/A T/T C/C A/G C/C A/A A/G C/C C/C G/G C/C A/A C/C G/G T/T C/C G/G G/G A/A C/C C/C G/G A/A A/A T/T C/C C/C G/G A/A T/T A/A A/A T/T A/T T/T T/T A/T G/G T/T T/T A/A T/T G/G T/T T/T A/A G/G C/C A/A G/G A/G A/G A/C A/A A/G C/C G/G T/T C/C C/T T/T A/A C/C G/G A/A A/G A/A T/T A/A G/G C/C C/C C/C G/G G/G G/G G/G T/T C/C T/T T/T A/A A/G C/C G/G T/T A/A A/A G/G G/G G/G G/G C/T A/A A/A A/A T/T T/T T/T C/C A/A G/G A/A T/T C/T G/G C/C A/A T/T T/T C/C T/T G/G T/T G/G A/A G/G A/A T/T C/C G/G C/C C/C G/G C/C C/C T/T G/G T/T T/T T/T A/A C/C C/C C/C T/T T/T G/G T/T A/A G/G C/G C/G C/C T/T C/C C/C A/A G/G G/G A/A C/C C/C T/T A/A G/G G/G T/T A/A A/A A/A T/T T/T A/A A/A T/T T/T C/C C/C G/G A/A C/C C/C C/C G/G C/C T/T T/T G/G A/A C/C G/G A/A G/G T/T C/C C/C C/C G/G G/G G/G C/C C/C T/T T/T G/G C/C C/C C/C G/G G/G T/T A/A T/T G/G T/T G/G T/T C/C T/T T/T T/T G/G A/A C/C A/A A/A T/T C/C G/G A/G C/C A/A C/C A/A G/G T/T G/G C/C C/C T/T G/G C/C A/A A/A C/C A/A T/T C/C C/C C/C A/A C/C C/C C/C G/G A/A A/A C/C T/T A/G G/G T/T G/T A/A C/C C/C C/C C/C T/T T/T C/C C/C C/C A/A T/T C/C A/A C/C C/C A/A T/T G/G G/G C/T G/G C/C A/A T/T T/T T/T T/T A/A C/C A/A G/G A/A C/C A/A A/A T/T T/T A/T
6-208-3	Ceq19(CeqEP29)		Name: Ceq19; fingerprint code: A/T G/T A/G A/G A/G A/G C/T C/G A/A A/A C/C C/C T/T G/G A/T T/T C/T C/C A/A C/C G/G A/G C/T C/T G/G C/C A/G A/G C/T C/C A/A A/C C/T C/C G/G C/C C/T A/G A/A A/A A/C A/G A/A T/T T/T C/C T/T G/G C/C C/C G/T G/G G/G T/T C/C G/G A/G C/T A/G G/T C/T A/A G/G A/A A/A A/A A/A A/A G/T A/G A/G C/C A/G C/G T/T A/G A/A C/C A/A A/A C/T G/G A/G A/C T/T T/T T/T C/T T/T A/A C/T A/G A/G C/C T/T A/A A/C A/G C/T A/G A/T C/C A/G A/G A/A C/T C/T A/G A/A A/G C/T C/T C/C A/G A/C A/T A/C A/G C/T A/T T/T T/T T/T A/G C/T C/C A/C G/T A/A A/T C/T A/G A/G C/C A/G G/G A/A G/G A/A A/A A/A C/C G/G T/T A/A T/T C/T A/G T/T A/A G/G A/G G/G A/A C/C A/A T/T T/T T/T A/A A/A A/A G/T C/C T/T C/T C/T G/G A/A T/T A/A C/C G/G A/G C/C C/G A/A A/G C/T G/G T/T G/G C/T G/T C/T C/T A/G C/C A/G A/T C/C G/T C/T G/G C/T C/T A/C G/T A/G C/C A/A T/T T/T G/G A/A T/T C/C T/T T/T C/C T/T T/T A/A A/A C/C C/T T/T A/A C/G C/G C/T A/A C/C A/A A/A A/G A/G C/G G/G T/T C/C C/T A/A C/C A/G A/G G/G A/C C/G G/T A/G A/G A/G C/T A/T A/A A/T C/T G/G G/G A/T C/T C/T C/T C/T A/G A/G C/G C/T A/C G/T A/A C/C T/T G/G G/G C/T A/G A/G C/G C/C C/G A/C T/T A/G A/G A/G C/T C/T T/T C/T G/G A/C A/C T/T A/G A/G C/C C/C A/T A/A C/T G/G C/T C/T C/T C/T C/T A/G A/T C/T A/G A/G A/T C/T A/A G/G A/A G/G A/A A/A A/G C/T G/T A/C C/G G/T A/A C/T G/G A/T A/C A/G C/T C/T A/A T/T G/G T/T T/T T/T A/A A/A A/A C/C T/T A/G G/T C/C G/T A/G C/T C/C C/C C/C C/C A/A C/T C/T C/T A/G G/T C/T A/G C/T C/G A/T G/T G/T A/G C/T A/G T/T T/T C/T C/T C/T A/A C/C G/G G/G C/C T/T T/T G/G T/T C/T T/T A/T
